# Synthesis and Biological Evaluation of Halogenated *E*-Stilbenols as Promising Antiaging Agents

**DOI:** 10.3390/molecules25235770

**Published:** 2020-12-07

**Authors:** Ester Sara Di Filippo, Letizia Giampietro, Barbara De Filippis, Marwa Balaha, Vincenzo Ferrone, Marcello Locatelli, Tiziana Pietrangelo, Angela Tartaglia, Rosa Amoroso, Stefania Fulle

**Affiliations:** 1 Department of Neuroscience Imaging and Clinical Sciences, Interuniversity Institute of Myology, University “G. d’Annunzio” of Chieti-Pescara, 66100 Chieti, Italy; es.difilippo@unich.it (E.S.D.F.); tiziana.pietrangelo@unich.it (T.P.); sfulle@unich.it (S.F.); 2 Department of Pharmacy, University “G. d’Annunzio” of Chieti-Pescara, 66100 Chieti, Italy; letizia.giampietro@unich.it (L.G.); marwa.balaha@pharm.tanta.edu.eg (M.B.); Vincenzo.ferrone@unich.it (V.F.); marcello.locatelli@unich.it (M.L.); angela.tartaglia@unich.it (A.T.); rosa.amoroso@unich.it (R.A.); 3 Department of Pharmaceutical Chemistry, Faculty of Pharmacy, Kafrelsheikh University, Kafr El Sheikh 33516, Egypt

**Keywords:** resveratrol, stilbenes, antioxidant activity, antiaging, *Log P*, HPLC-PDA

## Abstract

The increased risk of illness and disability is related to the age inevitable biological changes. Oxidative stress is a proposed mechanism for many age-related diseases. The crucial importance of polyphenol pharmacophore for aging process is largely described thanks to its effects on concentrations of reactive oxygen species. Resveratrol (3,5,4′-trihydroxy-*trans*-stilbene, RSV) plays a critical role in slowing the aging process but has a poor bioavailabity after oral intake. In this present work, a series of RSV derivatives was designed, synthesized, and evaluated as potential antioxidant agents. These derivatives contain substituents with different electronic and steric properties in different positions of aromatic rings. This kind of substituents affects the activity and the bioavailability of these compounds compared with RSV used as reference compound. Studies of *Log P* values demonstrated that the introduction of halogens gives the optimum lipophilicity to be considered promising active agents. Among them, compound **6** showed the higher antioxidant activity than RSV. The presence of trifluoromethyl group together with a chlorine atom increased the antioxidant activity compared to RSV.

## 1. Introduction

Aging, genetic and environmental factors play important roles in the onset of a wide range of chronic, metabolic, and neurodegenerative diseases [1]. Age-related disability and morbidity influence the quality of life; they are ultimately associated with an increased risk of death and serious consequences for the individual and society. Human aging is accompanied by a gradual increase of mental and physical impairment and thus, an increased risk of developing numerous diseases including cancer, diabetes, cardiovascular, musculoskeletal and neurodegenerative conditions [2].

Interestingly, it has been demonstrated that aging is a multifactorial phenomenon linked mainly to the production of free radicals [3,4]. Indeed, emerging studies suggest that peroxisomal function may also be altered with aging and contribute to the pathogenesis of a variety of diseases, including psoriasis, type-II diabetes (T2D), age-related macular degeneration (AMD), hypertension, Alzheimer’s disease (AD) and Parkinson’s disease (PD).[5,6] Moreover, it is knows that the Reactive Oxygen Species (ROS) accumulation, that is typical during ageing, could be responsible for both skeletal muscle impairments in elderly people [7] and in many age-related neurodegenerative diseases [8].

Many studies have been carried out in order to understand strategies to reduce the progression of aging [6]. Evidences prove that regular intake of berries, vegetables and fruits containing significant amounts of polyphenols is a potential way to improve the quality of life, using them like anti-aging [9,10,11]. In fact, in recent years, there has been a great deal of attention towards the molecular machinery relevant to age-related progression controlled through the exogenous intake of polyphenols that represent an epigenetic-modulating diet [9].

**Figure 1:**
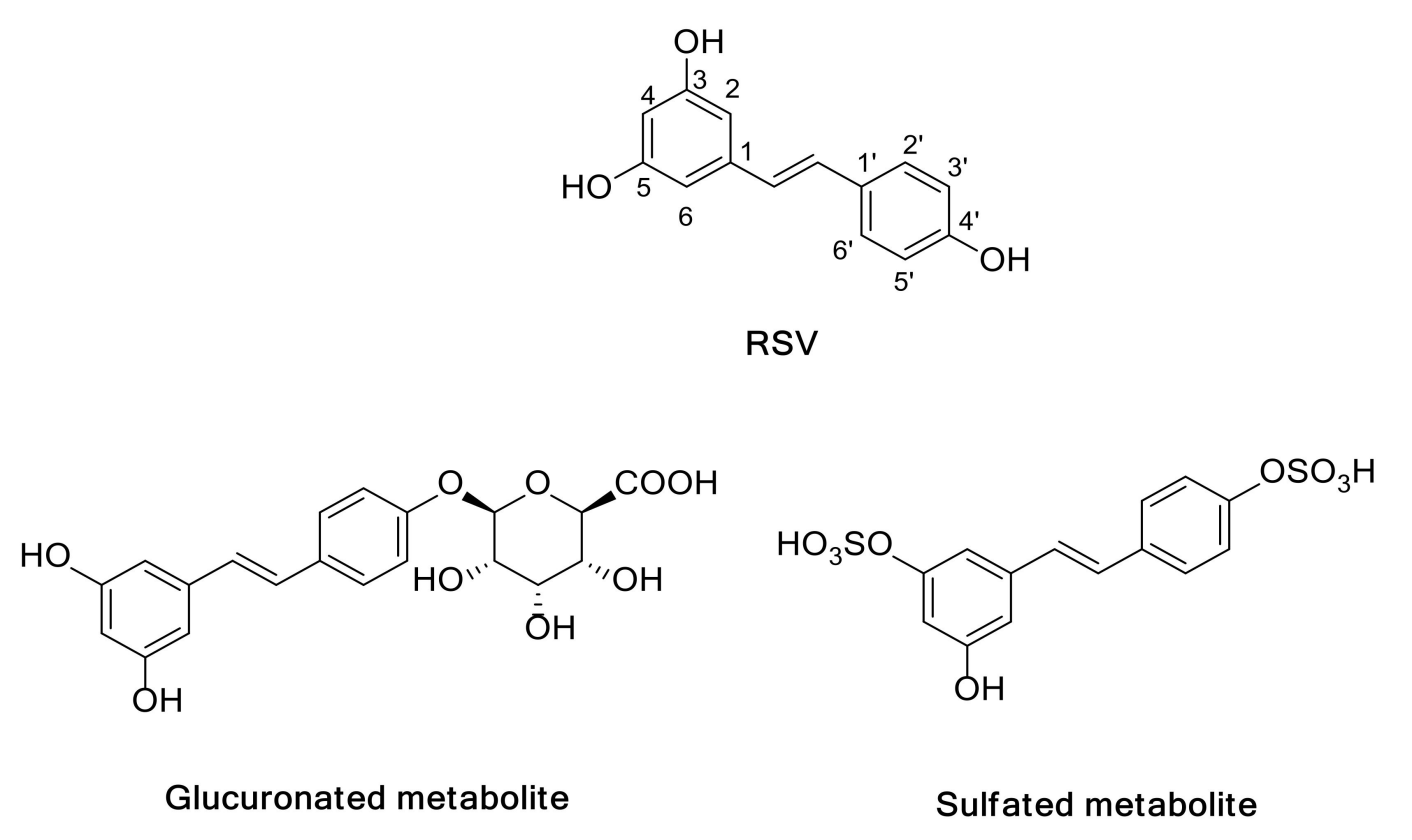
RSV and its glucuronated and sulfated metabolites.

Resveratrol (3,5,4′-trihydroxy-*trans*-stilbene, RSV, Figure 1) is a naturally occurring polyphenol with a stilbene skeleton found in at least 70 plant species, a number of which are dietary components including grapes, mulberries, and peanuts [12]. RSV has attracted much interest in the past decade because its well-known natural antioxidant [13,14] and cancer chemo-preventive activity [15,16,17]. There are several comprehensive reviews and scientific reports available focusing on its multiple pharmacological activities, such as anti-inflammatory, antimicrobial, neuroprotective [18,19] and cardioprotective effects [16,20,21]. RSV has been shown to inhibit the production of different reactive oxygen species (O_2_*^-^, H_2_O_2_, singlet oxygen and organic radicals) [22,23] and, in particular RSV has an O_2_*^-^ scavenging activity and also suppresses O_2_*^-^ generation by inhibiting xanthine oxidase (XO) activity [24,25]. RSV could improve myotube survival and prevent myotube atrophy in mature differentiated myotubes following glucose restriction (GR). While its effect on endothelial blood vessel cells, cancer cells, inflammatory processes and neurodegenerative events is well documented, little is known about the implication of RSV in differentiation processes, particularly in skeletal muscle cells [26].

Recently, the effects of RSV on mouse skeletal muscle derived cells (C2C12 cells) in either undifferentiated (myoblasts) or differentiated state (myotubes) have been reported [27,28,29,30]. RSV inhibits protein degradation and attenuates atrophy of skeletal muscle fibers [26,31].

The atrophy of skeletal muscle fibers is a process to which the skeletal muscle tissue physiologically undergoes during aging. This last one is a process closely related to an increase in oxidative stress due to the accumulation of free radicals. Indeed, it has been shown that high levels of ROS contribute to muscle cell death in aging with impaired regeneration and consequent disuse [32,33]. During adulthood, the muscle regeneration is guaranteed by adult muscle stem cells, the satellite cells. They are normally quiescent and when activated by physical, mechanical and/or harmful stimuli they activate, proliferate, and differentiate into new fibers or repair damaged ones. The cell line of murine myoblasts C2C12, derived from isolated satellite cells from regenerating muscle of adult mice [34], represents one of the most used cell lines for the study of skeletal muscle biology in vitro. The C2C12 cell line in culture provide an excellent model for the study of myogenic differentiation in vitro as this process is easily inducible through the composition of the culture medium and controlled by monitoring the parameters related to differentiation. After numerous steps in vitro, they undergo a replicative senescence that mimics aging in vivo. RSV showed an interesting benefit in skeletal muscle [35].

Unfortunately, RSV has been associated with poor bioavailability (less than 1%) specially due to extensive metabolism in the intestine and liver by glucuronidation and sulfation pathways results in unfavorable pharmacokinetic properties, consequently the application of RSV is greatly restricted [36,37,38]. Examples are shown in the Figure 1. The development of RSV derivatives is one of the most followed strategies to modulate its pharmacokinetic properties. The substitution of hydroxyls with groups with different electronic and steric properties in different positions leads to derivatives with improved bioavailability and often with higher activity [39].

The structural determinants of RSV, and in particular the hydroxyl groups, were studied in deep and their critical role in the antioxidant activity was highlighted [40,41]. The antioxidant activity of RSV is related to its hydroxyl (OH) groups; they are able to scavenge free radicals produced in vivo [42]. In particular, the 4′-position appears to be important in mediating the biological activity of RSV and stilbene containing derivatives with the (*E*)-conformation [41,43,44].

**Figure 2:**
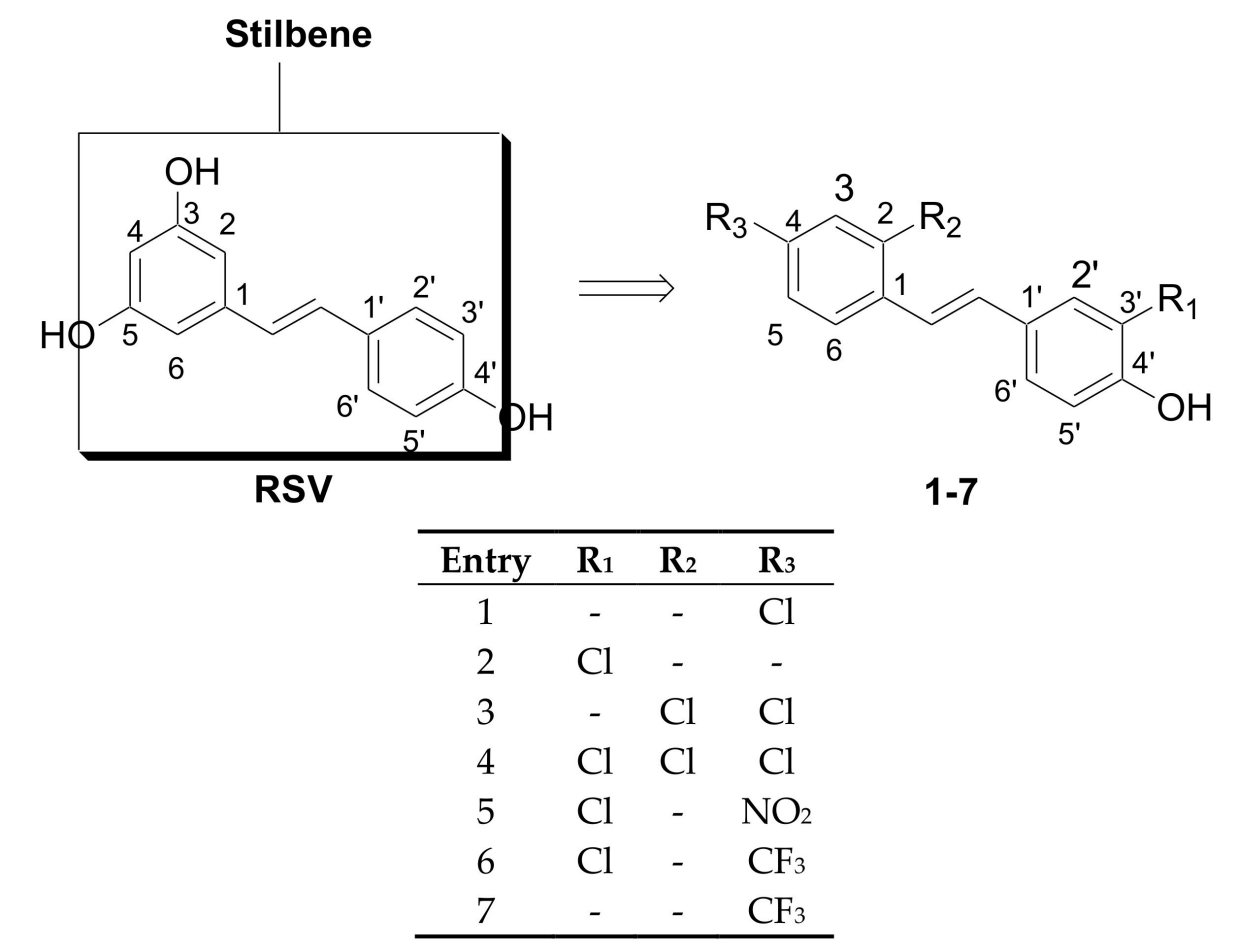
RSV and new derivatives (**1**–**7**).

In our recent researches, we have studied the different effect of para-substituent on 4-stilbenol ring on vitality of three pancreatic cancer cell lines and the effect of combination of stilbene moiety with alkanoic side chain on the vitality on C2C12 and MCF7 cells [16,45]. In continuation of our studies to give insight into the effect of different substituent on biological activity and given the potential of these molecules as therapeutic agents, we set out to investigate seven new stilbene derivatives and their structure-activity relationships. In this study, efforts have been directed towards exploring novel antioxidant agents by introducing different substituents on stilbene scaffold of RSV. We synthesized compounds **1**–**7** in which the 4′-hydroxyl group was kept unchanged, given its importance for biological activity [40,41].Considering the positive effect of halogens as bromo-, iodo- and trifluoromethyl group on the free radical activity of RSV derivatives, [46] we explored the influence on the activity of chlorine in position 3′ and/or 2,4-position instead of the hydroxyls in 3,5 position of RSV in compounds **1**–**4.** In compounds **5**–**7** nitro or trifluoromethyl groups in 4-position were introduced. The chemical structures are depicted in Figure 2. Some of them were synthesized as intermediate of different final products [47]. In view of the unfavorable pharmacokinetic profile of RSV, we expected that the introduction of one or more chlorine atoms could generate a new class of stilbene compounds with the improved pharmacokinetic properties and ADME profile. This is because these new derivatives are less favorable substrate of glucuronidase and sulfatase due to the presence of a single hydroxyl group for conjugation reactions, which further lead to a better metabolic stability than RSV [48,49,50]*. Log P* calculations were employed to study the lipophilicity of the designed molecules and the possible effect on the antioxidant profile of the scaffold. All compounds were evaluated for their ability to modulate the C2Cl2 cell line vitality and **6** for its antioxidant activity.

## 2. Results

### 2.1. Chemistry

**Scheme 1:**
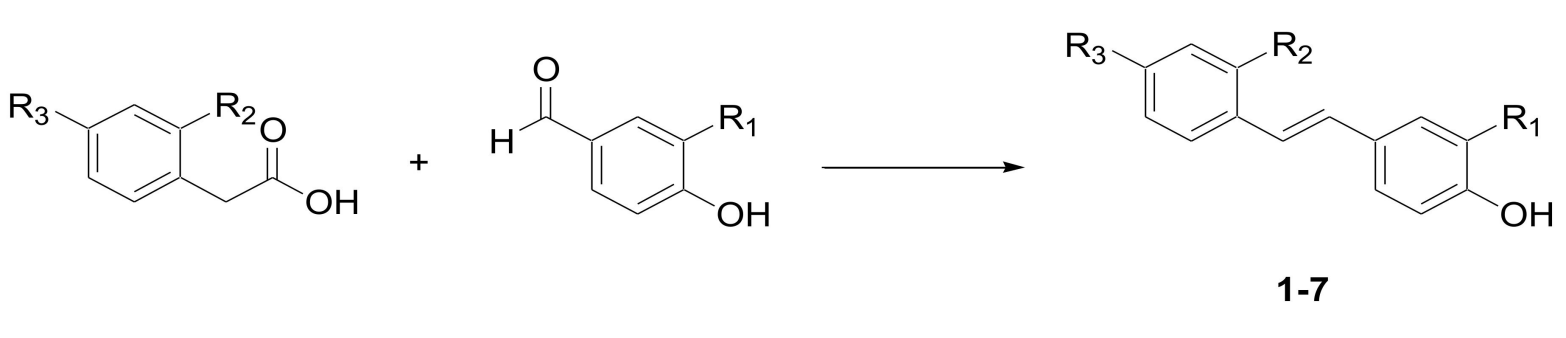
Synthesis of compounds **1**–**7**. Reagents and conditions: suitable 4-hydroxybenzaldehyde (1.0 eq.), suitable aryl acetic acid (1.2 eq.), piperidine (2.5 eq.), 130 °C, 4–24 h.

The synthesis of compounds **1**–**7** was carried out following reported procedures [16,47]. The proper 4-hydroxybenzaldehyde and the suitable aryl acetic acid were mixed in the presence of piperidine at 130 °C. The usual aqueous work-up and purification using silica gel column chromatography produced the phenols **1**–**7**. The synthetic route is in Scheme 1 (see Section 3, Materials and Methods for the detailed procedures).

Confirmation of the structures and purity of all compounds were obtained from ^1^H and ^13^C-NMR and the geometry of the double bond was established by *J*-values range from 15 to 16 Hz of *trans*-olefinic proton respect of *cis*-stilbene olefinic protons from 7.4 to 8.6 Hz reported in literature [51]. The purity of each compounds was evaluated by HPLC-PDA procedure during the *Log P* analyses (see Supplementary Materials for the detailed procedures), considering the counter plot results and the peak area values corresponding to analyte maximum wavelength.

### 2.2. Biology

**Figure 3:**
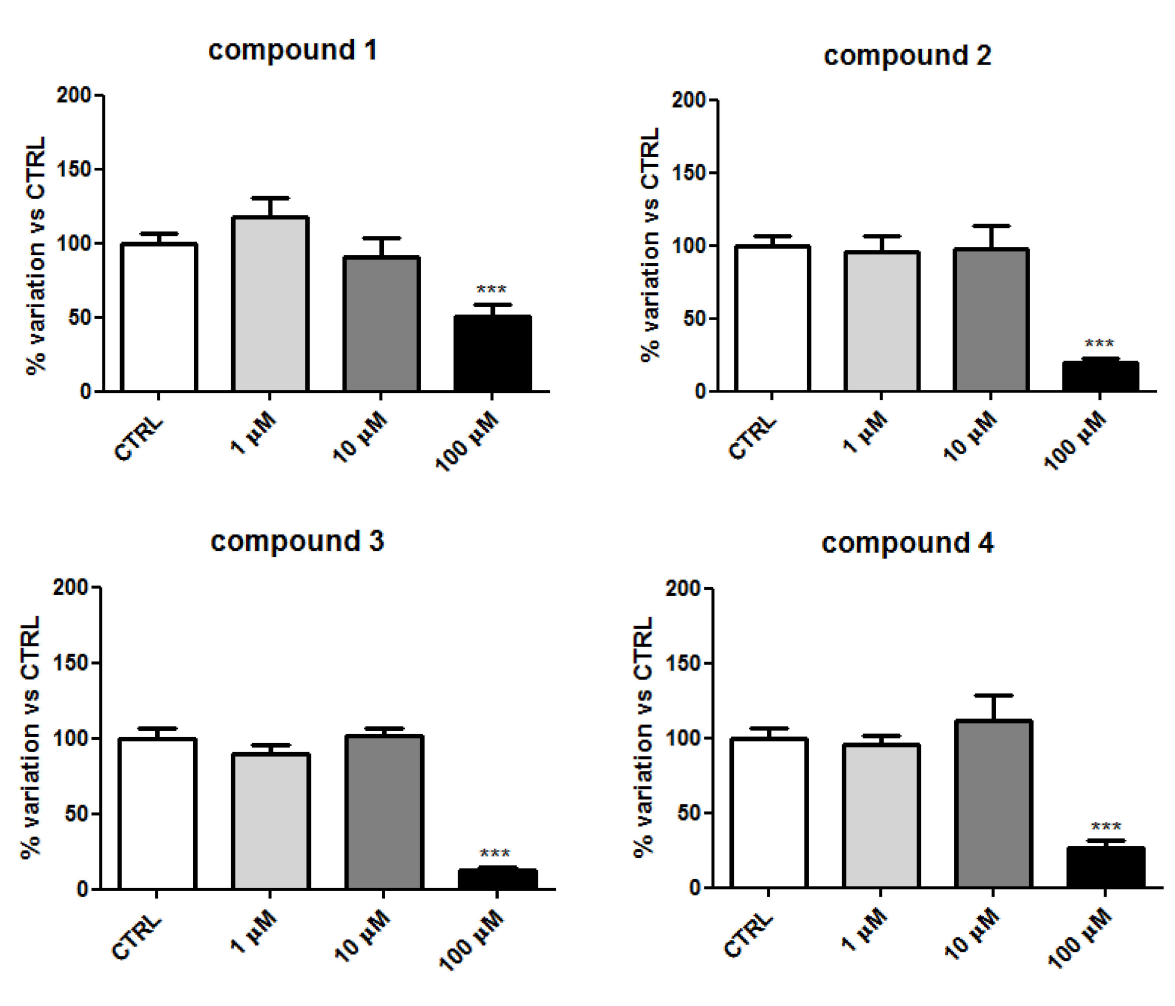

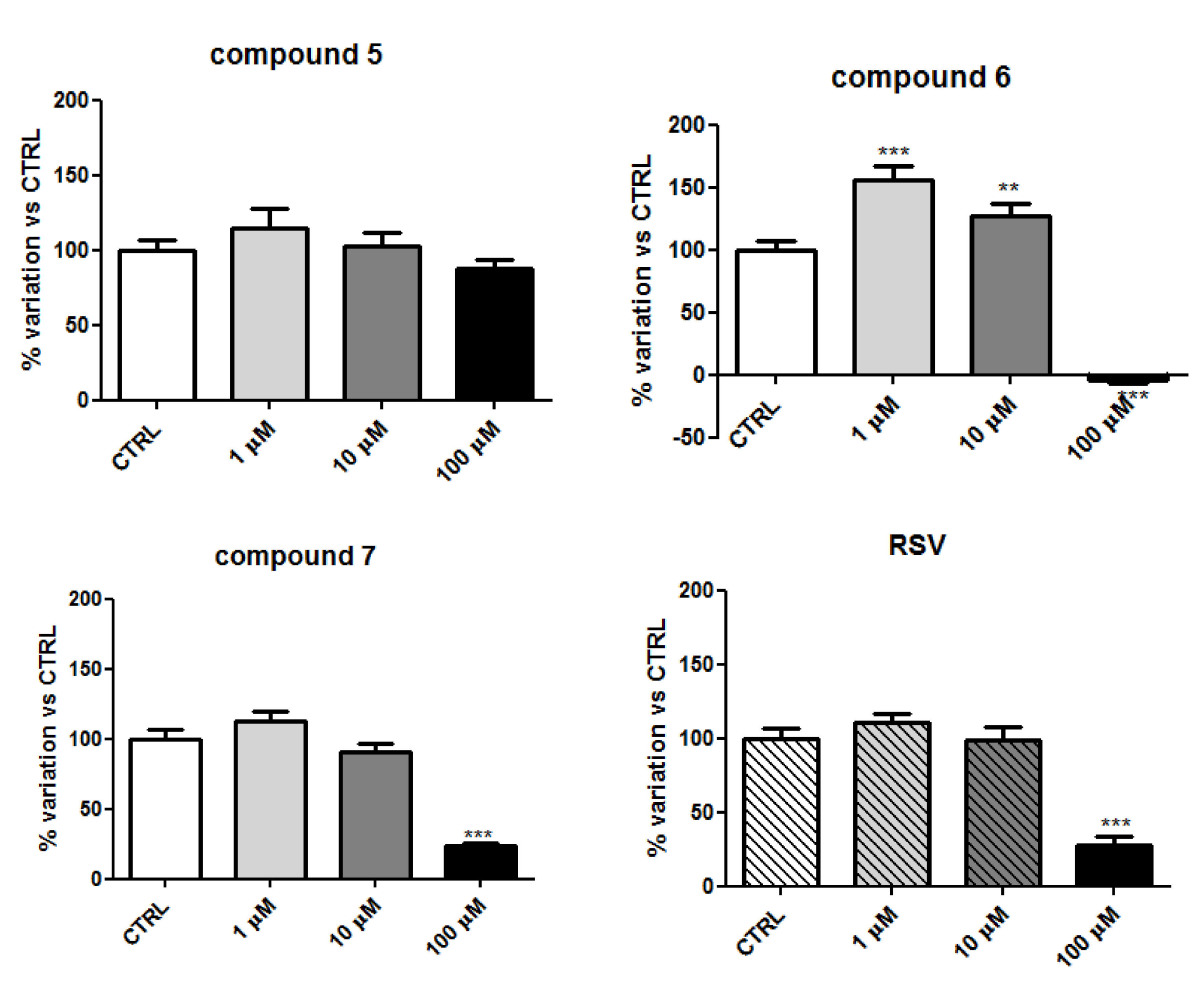
Dose-response effects of compounds (**1**–**7**) and RSV in C2C12 cells after 24 h. MTT reduction assay in C2C12 cells in the presence of (**1**–**7**) together with RSV. The cells were incubated with increasing concentrations of the compounds (1, 10, 100 µM) for 24 h. After this period, cell viability was quantified by measuring MTT reduction. CTRL: control, cells w/o compounds. Data are expressed as % variation vs CTRL. The means ± SEM derived from 3 different experiments (each with *n* = 8) and were analysed using unpaired *t*-tests. *** *p* < 0.0005, ** *p* < 0.001.

**Figure 4:**
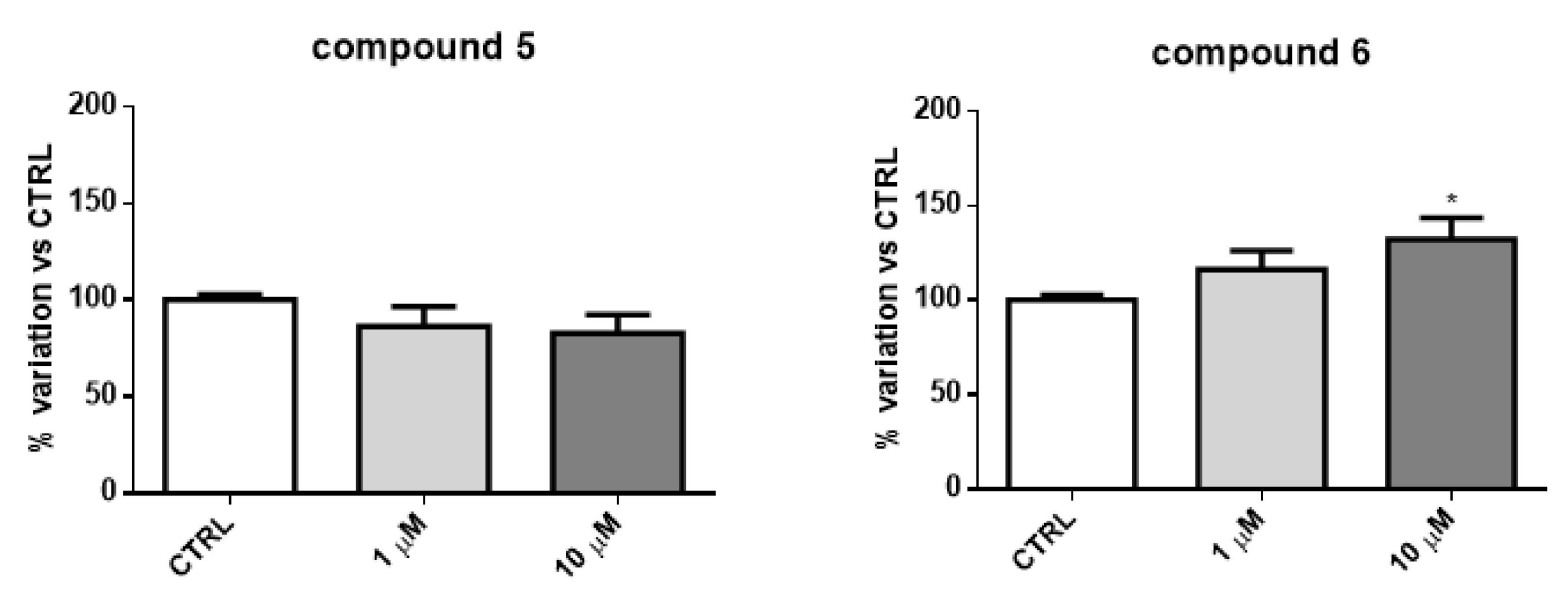

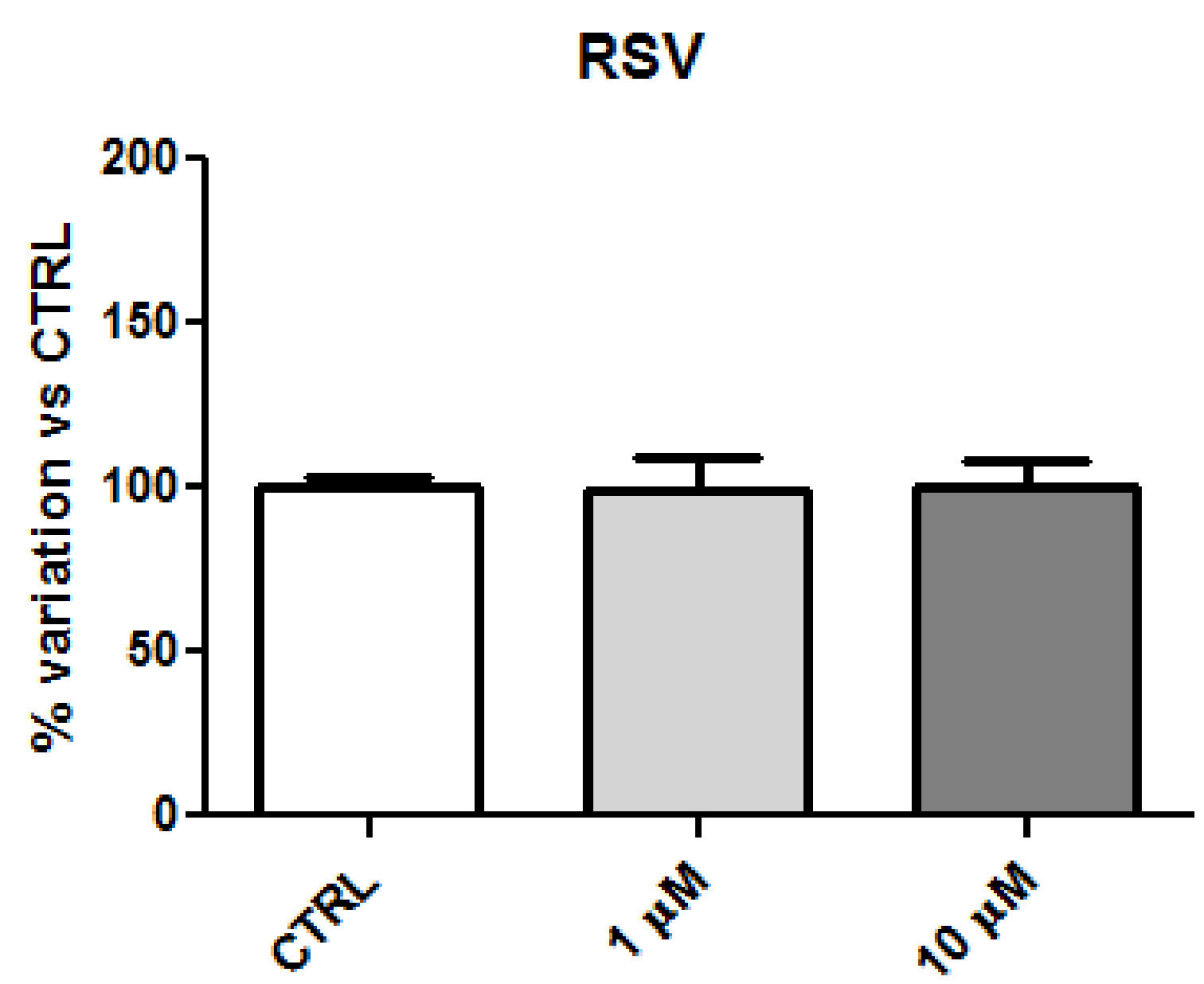
Dose-response effects of compounds **5**, **6** and RSV in C2C12 cells after 48 h. MTT reduction assay in C2C12 cells in the presence of compounds **5**, **6** and RSV. The cells were incubated for 48 h at different concentration of the compounds (1, 10 µM). After this period, cell viability was quantified by measuring MTT reduction. CTRL: control, cells w/o compounds. RSV: resveratrol. Data are expressed as % variation vs. CTRL. The means ± SEM derived from 3 different experiments (each with *n* = 8) and were analysed using unpaired t-tests, * *p* = 0.0345.

It is important to verify that a compound that may have antioxidant capacity does not negatively interfere with cell viability. The oxidative stress plays one of the key roles in cell’s life path [4]. For these reasons, to evaluate the possible cytotoxic effects of compounds **1**–**7** and their effect on the cell proliferative capacity and to define a suitable concentration range, all the new synthesized compounds **1**–**7** were evaluated for their cytotoxic activity at three different concentrations (1, 10 and 100 μM) via standard MTT assay, using C2C12 cell line (Figure 3) [52,53]. Due to well-known antioxidant and neurodegenerative senescence prevention abilities in addition to enhancing the beneficial effects of physical exercise on the skeletal muscles of the elderly, RSV was used as reference compound [54,55]. The results showed that after 24 h of treatment at concentration of 1 and 10 μM, **1**–**7** are not toxic for the cells respect to RSV at the same concentrations, even though they appeared to promote the cell proliferation without a common profile. Antiproliferative activity was observed at 100 μM for all compounds, except for compound **5**. Only compound **6** showed a significant increase of the cell vitality at 1 and 10 μM but at 100 μM it drastically reduced the vitality. For this reason, we performed the same assay also at 48 h for the compounds **5**, **6** and RSV (Figure 4). **5** showed non-significant dose-dependent decrease of vitality, while compound **6** confirmed an enhanced vitality also at 48 h with reversal of the trend, moderate effect at 1 μM and higher effect at 10 μM. RSV did not change the effects respect to the control (CTRL). In addition, because **1**–**7** were dissolved in DMSO, we also tested the cells with only DMSO for 24 h (see Section 3, Materials and Method) confirming that chosen concentrations did not interfere with cellular vitality (data not shown).

**Figure 5:**
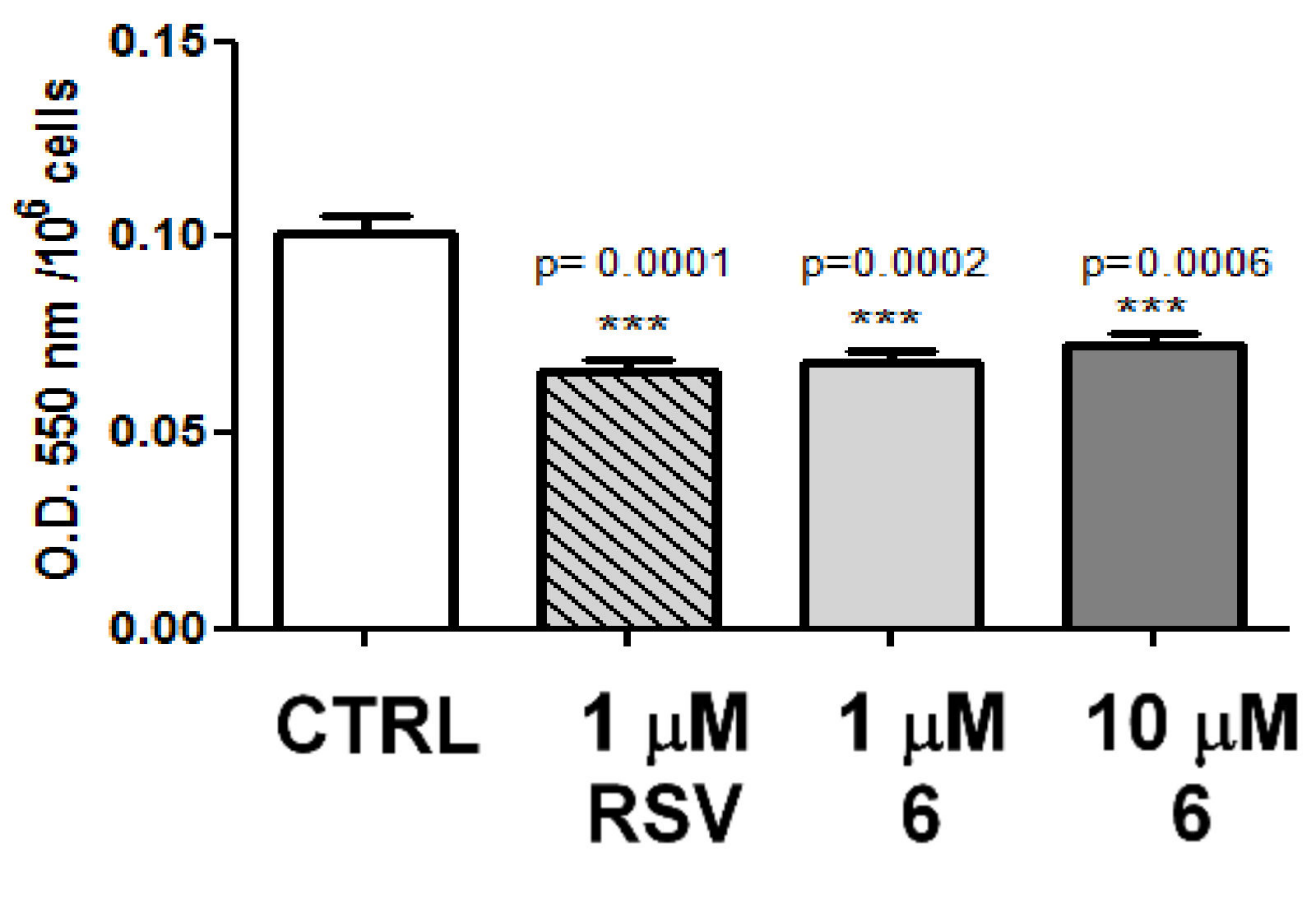
Superoxide anion detection in C2C12 cells treated with the compound **6** and RSV. NBT assay after 24 h of incubation of C2C12 cells in the presence of compound **6** and RSV for O^•−^_2_ detection. CTRL: control, cells w/o compounds. The means ± SEM derived from 3 different experiments (each with *n* = 5) and were analysed using unpaired *t*-tests, control vs samples, *** *p* < 0.001.

Since only compound **6** showed a significant increase of proliferative capacity at 1 and 10 μM and maintained an improvement effect at 48 h, it was selected for the evaluation of its antioxidant capacity by using two different methods, namely, NBT and H_2_DCFDA assay. At first, the antioxidant effect of compound **6** was assessed by NBT assay (Figure 5). The NBT assay is based on the reduction of NBT into formazan by superoxide anion O_2_^-^. The formazan concentration is determined using a spectrophotometer, such that more formazan indicates that more O_2_^-^ had reduced NBT. The concentration of superoxide anion radical in the cells was determined by spectrophotometric method and is based on the reduction of Nitro blue tetrazolium chloride to Nitro blue-formazan in the presence of O_2_^-^. The capturing of O_2_^-^ antioxidant compounds inhibits the formation of NBT and consequently decreases its absorption at 550 nm.

**Figure 6:**
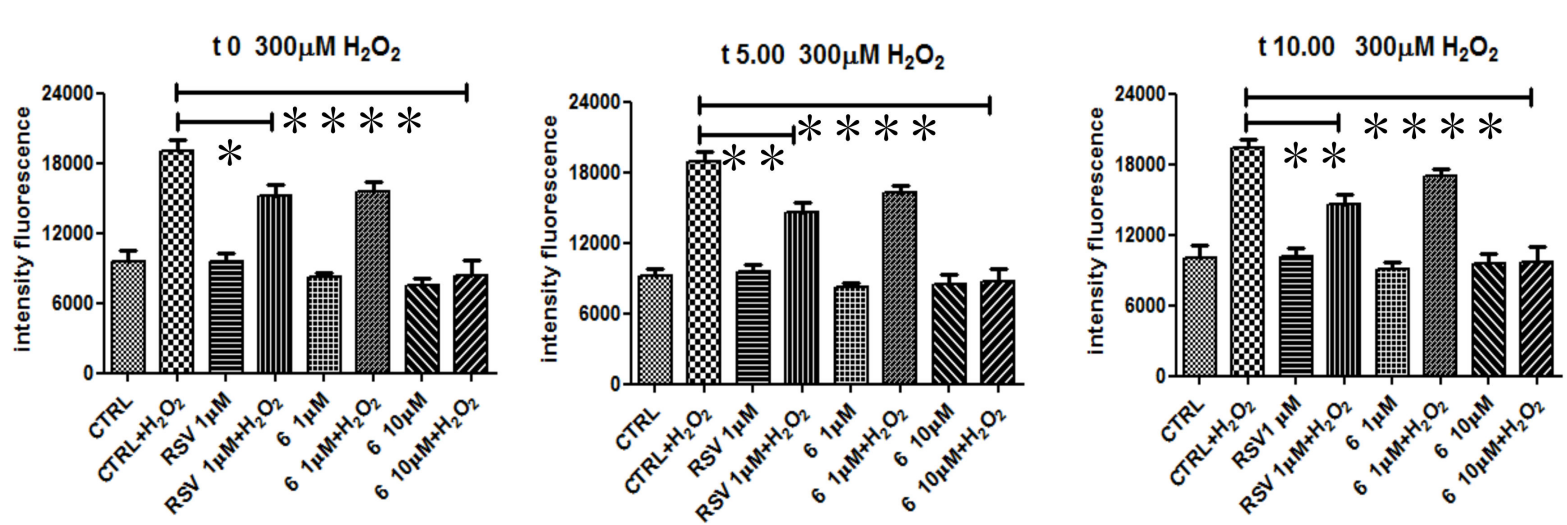
Antioxidant capabilities of the compound **6** and RSV in C2C12 cells treated with the oxidant insult H_2_O_2_. Quantitative analyses of ROS expression. ROS measurement by H_2_DCF-DA fluorescence in C2C12 cells exposed to compound **6** or RSV 24 h before and during a 10-min incubation period with 300 µM H_2_O_2_ concentrations. In this figure the fluorescence at three points (t0, t5 and t10) during the time-course are reported. The fluorescence intensity represents the mean of values registered during 10 min ± SEM derived from 3 different experiments (each with *n* = 8). * *p* < 0.05, ** *p* < 0.01, **** *p* < 0.0001.

Interestingly, a significant decrease of O_2_^-^ levels after 24 h of incubation in the presence of compound **6** at 1 and 10 μM, comparable to that of RSV, was observed. This result clearly evidences the ability of compound **6** to scavenge the superoxide anion O_2_^-^ in cells. Moreover, it is known that also H_2_O_2_ directly produces a high quantity of ROS, further damaging cells. For this reason, in the following experiment (Figure 6), we evaluated the activity of compound **6** in C2C12-lesionated with H_2_O_2_.

Indeed, the antioxidant activity of compound **6** was also evaluated using an H_2_DCFDA assay (Figure 6). This assay is one of the most widely used techniques for directly measuring the redox state of a cell [56,57,58]. In order to accurately measure ROS and the cellular capability to counteract the H_2_O_2_ insult, we used cell-permeable fluorescent and chemiluminescent probes. The fluorescence intensity is proportional to the ROS levels within the cell cytosol and it is in inverse relation to the antioxidant capacity of the cells themselves. The ability to restore the basal level of fluorescence are reported in Figure 6, after 0.5 and 10 min following oxidant stimulus. During the assays, the cells, after the addition of 300 µM H_2_O_2_, can reduce the ROS levels in the presence of RSV and compound **6**, compared to the CTRL showing a significant antioxidant activity. In particular, we observed that the oxidant insult, represented by H_2_O_2_, was strongly counteracted by the tested compound **6** at 10 μM. This result remains stable throughout the time-course. The compound **6**, at this concentration, restoring the fluorescence values close to those of cells without H_2_O_2_, showed a significantly higher antioxidant capacity with respect to RSV. These results confirmed the high antioxidant capacity of the new synthesized compound against two ROS harmful to cellular life. Moreover, compound **6** showed potentially greater protective properties respect to RSV; these data suggest that it could be a novel candidate for in vivo studies to further investigate its effects in those pathologies characterized by oxidative stress.

### 2.3. Log P Analysis

The successful studies of drug candidates require optimization of molecular parameters simultaneously using different methods, both experimentally and theoretically [49]. *Log P* study is of significant interest in biology, pharmacology or medicine [59,60,61]. The lipophilicity was first predicted computationally (*Log P*) with the ChemBioDraw Ultra 12.0 software. Then, the results were experimentally confirmed by using HPLC-PDA procedure in order to avoid the drawbacks related to interferences and/or degradation product in classical shake-flask assay [59]. The *Log P* of reference compound RSV was also experimentally determined, and the data were found to be in accordance with the literature [62].

**Figure 7:**
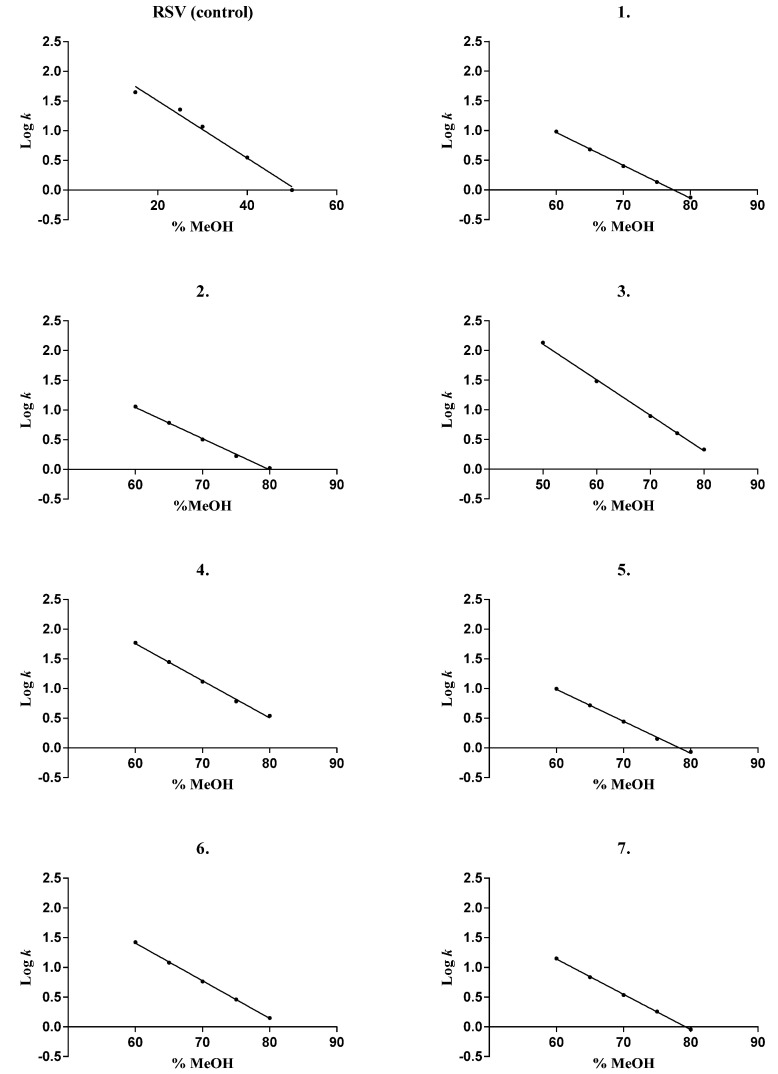
Linear regression correlation between % MeOH and Log k for (**1**–**7**) and RSV.

For each compound, the retention time was reported at different percentages of mobile phases (Table 1). See Materials and Methods and Supplementary Materials for the detailed procedures. The capacity factors (*k*) of new compounds were calculated as follow and data are reported in Table 2.

(1) $$k=\;\frac{t_{R}-\;t_{M}}{t_{M}}$$

where *t_R_* is the retention time of the sample and *t_M_* is the retention time of blank (methanol). Subsequently, the logarithm of capacity factor (Table 3) was plotted against percentages of organic mobile phase (methanol) and *Log P* was extrapolated as 0% composition of MeOH, as reported in Figure 7 that shows the graph with *Log k* in *y*-axis and percentages of MeOH in *x*-axis.

The experimental *Log P* value calculated for each compound was compared with *Log P* obtained using ChemBioDraw Ultra 12.0 software. The results are shown in Table 4.

### 2.4. Discussion

It is known that the proliferative capacity is negatively influenced by oxidative stress and the presence of ROS, as we have already demonstrated [32,57,58]. These radicals are normally detoxified by endogenous scavenger systems, assisted by exogenous antioxidants. Natural polyphenols are considered ROS scavengers reacting with free radical and protecting lipid and DNA from oxidative damage. Down regulation of free radical levels is expected to be beneficial for cellular vitality and in reducing many common age-related diseases [63].

Our results confirm the new compound **6** as a molecule with an interesting antioxidant potential and without cytotoxic effects. It could therefore be hypothesized that this compound could also be able to exert positive effects on the proliferation enhancement, as consequence of its antioxidant properties, even more evident at 10 μM. This fact could be related to the ability of **6** to scavenge ROS due the presence of a 4-CF_3_ and the 3′-Cl in *ortho* to the 4′-hydroxyl. This is in line with studies that reported how the antioxidant activity remarkably increases with increasing the electron-rich environment in the molecules [14]. In particular, the 3′-ring position is critical for radical scavenging, as previously described: the influence of neighboring group to 4′-hydroxyl could promote the formation of intramolecular hydrogen bond stabilizing the hydroxyl group in the 4′ position and makes it more active [14,44]. Similarly, we assume that the electronic effect of the 3′-chlorine atom might improve the scavenger ability of 4′-hydroxyl again in this case.

The obtained results show that there is a small difference between the *Log P* calculated by experimental method (HPLC) and theoretical *Log P* estimated by software. The presence of three hydroxyl groups restricts the bioavailability of RSV in vivo as its oral bioavailability is almost zero resulting with only trace amount of unchanged RSV in the systemic circulation due to rapid and extensive metabolism and the consequent formation of various metabolites as glucuronides and sulfates. Dimethyl ether derivative of RSV, pterostilbene was found to be more metabolically stable and usually exhibited stronger pharmacological activities than that of RSV. This returns to that pterostilbene is less favorable substrate of glucuronidase and sulfatase as only a single hydroxyl group available for conjugation reactions [50,64]. These findings ensure that our newly synthesized compounds have bioavailability more superior than that of reference compound. This is associated with the replacement of two hydroxyl groups with more lipophilic substituents-in different positions. For this reason and starting from biological data, we were interested in knowing the *Log P* values of our compounds. All compounds showed values of *Log P* (Figure 6) superior to RSV. This is associated with the presence of two or three chlorine atoms or a trifluoromethyl group. All compounds have *Log P* values around 5.0, with exception of the compound **4** which has *Log P* value superior of 5.0, making six out of seven compounds likely orally active drugs in humans. In particular, the compound **6** is the most active one. The physicochemical characterization of these compounds in terms of *Log P* provided here is only informative for future optimization to gain more information about brain penetration, clearance rate and metabolic stability.

## 3. Materials and Methods

### 3.1. Chemistry

Melting points were determined with a Buchi Melting Point B-450. ^1^H and ^13^C-NMR spectra were recorded on a Varian Mercury 300 spectrometer. Proton chemical shifts were referred to the TMS internal standard. Chemical shifts are reported in parts per million (ppm, δ units). Coupling constants are reported in units of Hertz (Hz). Splitting patterns are designed as s, singlet; d, doublet; t, triplet; q quartet; qnt quintet; dd, double doublet; m, multiplet; b, broad. Infrared spectra were recorded on a FT-IR 1600 Perkin Elmer. All commercial chemicals and solvents are reagent grade and were used without further purification unless otherwise specified. All reactions were carried out with the use of the standard techniques and were monitored by thin-layer chromatography on silica gel plates (60F-254, E. Merck, Merck Group, Darmstadt, Germany) and visualized with UV light.

#### General Procedure for the Preparation of Phenols **1**–**7**

A stirred mixture of piperidine (2.5 eq., 1.01 mL, 10.23 mmol), 4-hydroxybenzaldeyde or 4-hydroxy-3-chlorobenzaldeide (1.0 eq., 500 mg, 4.09 mmol), and properly phenylacetic acid (1.2 eq., 4.91 mmol) was heated gradually to 130 °C and allowed to react for 4–24 h, the residue was cooled at room temperature and partitioned between EtOAc (3 × 30 mL) and H_2_O (20 mL). The organic phase was dried over Na_2_SO_4_ and concentrated under reduced pressure to yield the crude product that was purified by column chromatography (eluent CH_2_Cl_2_/MeOH 95:5 or cyclohexane/ethylacetate 6:4) giving the pure phenols **1**–**7 [16]**.

4-[(*E*)-2-(4-Chlorophenyl)vinyl]phenol, **1**. White solid, 0.16 g (56%); mp 184–185 °C. ^1^H-NMR (CD_3_OD) δ 6.83 (d, *J* = 9.0 Hz, 2H, C*H*_Ar_), 6.95 (q, *J* = 20.7 Hz, 2H, C*H*=C*H*), 7.28 (d, *J* = 9.0 Hz, 2H, C*H*_Ar_), 7.38 (d, *J* = 9.0 Hz, 2H, C*H*_Ar_), 7.40 (d, *J* = 9.0 Hz, 2H, C*H*_Ar_); ^13^C-NMR (CD_3_OD) δ 115.33 (*C*H_Ar_), 127.36 (*C*H_Ar_), 120.37 (*C*H=CH), 124.11 (CH=*C*H), 136.93 (*C*_Ar_), 132.22 (*C*_Ar_), 157.54 (*C*_Ar_); IR (neat) 3267, 2728, 1606, 1513, 1245 cm^−1^.

2-Chloro-4-[(*E*)-2-phenylvinyl]phenol, **2.** Brown solid, 0.99 g (42%); mp 126–127 °C. ^1^H-NMR (CDCl_3_) δ 7.03 (d, *J* = 9.0 Hz, 1H, C*H*_Ar_OH), 7.48–7.50 (m, 3H, C*H*_Ar_), 7.25–7.39 (m, 5H, C*H*_Ar_ + C*H*=C*H*); ^13^C-NMR (CDCl_3_) δ 116.68 (*C*H_Ar_), 120.53 (*C*_Ar_), 126.65 (*C*H_Ar_), 126.91 (*C*H_Ar_), 127.04 (*C*H_Ar_), 127.12 (*C*H_Ar_), 127.88 (*C*H=CH), 128.19 (CH=*C*H), 128.97 (*C*H_Ar_), 131.60 (*C*_Ar_); IR (neat) 3513, 3435, 3022, 1588, 1497 cm^−1^.

4-[(*E*)-2-(2,4-Dichlorophenyl)vinyl]phenol, **3.** Yellow solid, 0.58 g (23%); mp 119–120 °C. ^1^H NMR (CDCl_3_) δ 6.86 (d, *J* = 8.7 Hz, 2H, C*H*_Ar_OH), 7.08 (d, *J* = 16.2 Hz, 1H, C*H*=CH,), 7.22 (m, 2H, C*H*_Ar_), 7.29 (d, *J* = 14.1 Hz, 2H, CH=C*H*,), 7.39–7.46 (m, 2H, C*H*_Ar_), 7.59 (d, *J* = 8.4 Hz, 1H, C*H*_Ar_); ^13^C-NMR (CDCl_3_) δ 115.70 (*C*H_Ar_), 121.47 (*C*H_Ar_), 126.90 (CH=*C*H), 127.25 (*C*H_Ar_), 128.38 (*C*H_Ar_), 129.49 (*C*H_Ar_), 129.74 (*C*_Ar_), 131.14 (*C*H=CH), 133.57 (*C*_Ar_), 134.27 (*C*_Ar_), 155.78 (*C*_Ar_); IR (neat) 3240 (broad), 1592, 1443, 1231 cm^−1^.

2-Chloro-4-[(*E*)-2-(2,4-dichlorophenyl)vinyl] phenol, **4.** Brown solid, 0.121 g (41%); mp 135–136 °C; 1H-NMR (CDCl_3_) δ 6.83 (d, *J* = 8.7 Hz, 1H, C*H*_Ar_), 6.98 (dd, 2H, *J* = 18.9 Hz, C*H*=CH), 7.03 (s, 1H, C*H*_Ar_), 7.25 (t, *J* = 1.8 Hz, 1H,), 7.4 (d, *J* = 2.4 Hz, 1H, C*H*_Ar_), 7.50 (d, *J* = 2.4 Hz, 1H, C*H*_Ar_), 7.56 (d, *J* = 8.7 Hz, 1H, C*H*_Ar_); ^13^C-NMR (CDCl_3_) δ 116.08 (*C*H_Ar_), 116.49 (CH=*C*H), 119.67 (*C*_Ar_), 126.78 (*C*H=CH), 127.10 (*C*H_Ar_), 127.91 (*C*H_Ar_), 128.27 (*C*H_Ar_), 128.53 (*C*H_Ar_), 129.48 (*C*H_Ar_), 132.19 (*C*_Ar_), 132.97 (*C*_Ar_), 135.78 (*C*_Ar_), 149.84 (*C*_Ar_); IR (neat) 3507 (broad), 1595, 1467, 1262 cm^−1^.

2-Chloro-4-[(*E*)-2-(4-nitrophenylvinyl]phenol, **5.** Yellow solid, 0.33 g (30%); mp 201.0–203.0 °C; ^1^Η-NMR (CDCl_3_) δ 5.67 (s, 1H, broad, OH), 7.01 (d, 1H, *J* = 25.5, C*H*=CH), 7.031 (s, 1 H, C*H*_Ar_), 7.14 (d, 1H, *J* = 16.5, CH=C*H*), 7.38 (d, 1H, *J* = 8.1, C*H*_Ar_), 7.54 (d, 1H, *J* = 1.8, C*H*_Ar_), 7.59 (d, 1H, *J* = 8.7, C*H*_Ar_), 8.21 (d, 1H, *J* = 8.1, C*H*_Ar_); ^13^C-NMR (CDCl_3_) δ 116.63 (*C*H_Ar_), 120.66 (*C*_Ar_), 123.05 (*C*H=CH), 124.20 (*C*H_Ar_), 125.47(*C*_Ar_), 126.72 (*C*H_Ar_), 127.33 (*C*H_Ar_), 127.36 (*C*H_Ar_), 129.81 (CH=*C*H), 131.45 (*C*_Ar_), 148.76 (*C*_Ar_); IR (neat) 3507 (broad), 1595, 1467, 1262 cm^−1^.

2-Chloro-4-{(*E*)-2-[4-(trifluoromethyl)phenyl]vinyl}phenol, **6.** Yellow solid, 0.38 g (31%); mp 117.0–118.4 °C; ^1^H-NMR (CDCl_3_) δ 5.62 (s, 1H, OH), 6.97 (d, *J* = 16.5 Hz, 1H, C*H*=CH,), 7.06 (d, *J* = 16.2 Hz, 1H, C*H*=CH,), 7.02 (d, *J* = 8.1 Hz, 2H, C*H*_Ar_), 7.37–7.34 (m, 1H, C*H*_Ar_), 7.44 (d, *J* = 1.8 Hz, 2H, C*H*_Ar_), 7.55 (d, *J* = 8.7 Hz, 2H, C*H*_Ar_), 7.61 (d, *J* = 8.7 Hz, 2H, C*H*_Ar_); ^13^C-NMR (CDCl_3_) δ 116.49 (*C*H_Ar_), 120.37 (*C*_Ar_), 122.58 (*C*_Ar_), 125.63 (m, *C*H_Ar_), 126.32 (*C*H_Ar_), 126.43 (*C*H_Ar_), 127.01 (*C*H=CH), 127.05 (*C*H=CH), 129.33 (*C*_Ar_), 130.61 (*C*_Ar_), 147.97 (*C*_Ar_), 151.26 (*C*_Ar_).

4-{(*E*)-2-[4-(Trifluoromethyl)phenyl]vinyl}phenol, **7.** White solid, 0.31 (23%); mp 161–163 °C. ^1^H-NMR (Acetone-*d6*) δ 6.88 (d, *J* = 8.7 Hz, 2H, C*H*_Ar_), 7.13 (d, *J* = 16.5 Hz, 1H, C*H*=CH), 7.34 (d, *J* = 16.5 Hz, 1H, CH=C*H*), 7.52 (d, *J* = 8.7 Hz, 2H, C*H*_Ar_), 7.66 (d, *J* = 9.0 Hz, 2H, C*H*_Ar_), 7.75 (d, *J* = 8.1 Hz, 2H, C*H*_Ar_); ^13^C-NMR (Acetone-*d6*) δ 115.62 (*C*_Ar_), 123.83 (*C*H=CH), 125.37–125.53 (m, F_3_*C*), 126.46 (*C*_Ar_), 128.36 (*C*_Ar_), 128.46 (*C*_Ar_), 131.38 (*C*_Ar_), 142.05 (*C*_Ar_), 157.91 (*C*_Ar_); IR (neat) 3614, 3419, 3271, 1599, 1511, 1327, 1254, 1181, 836 cm^−1^.

### 3.2. Biology

#### 3.2.1. Cell Culture Medium Preparation

Prompted by the results obtained for compound **6** as a scavenge ROS due the presence of halogenated groups, we used the widely C2C12 mouse cells as a myogenic model, to study its antioxidant effects following both the evaluation of superoxide anion radical concentration and chemical insults, such as from H_2_O_2_. C2C12 cells (CRL 1772; American Type Culture Collection, Rockville, MD, USA) were grown in Dulbecco’s modified Eagle’s medium (DMEM; Euroclone, Pero, Italy) containing 20% foetal bovine serum (Euroclone), 200 mM L-glutamine (Euroclone) and 100 IU mL^−1^ penicillin and 100 μg mL^−1^ streptomycin (Euroclone). The cells were maintained at 37 °C in a 5% CO_2_ humidified atmosphere [52].

#### 3.2.2. MTT Assay

Cellular proliferation was tested using a colorimetric assay of 3-[4,5-dimethylthiazol-2-yl]-2,5-diphenyltetrazolium bromide (MTT). We seeded 2000 cells/well in a final volume of 200 μL growth medium in 96-well plates. After 24 h the cells were stimulated with or w/o the newly synthesized compounds for 24 h, or, where indicated, for 48 h. We tested seven new synthesized compounds together with RSV reference at different concentration (1, 10, 100 μM). All compounds were dissolved in DMSO and the experimental dilutions used were made using cellular growth medium. For this reason, the cells were also incubated for 24 h with the DMSO at the same concentration used for the compounds. Incubation was terminated by adding 20 μL of MTT solution (5 mg mL^−1^ in phosphate-buffered saline (PBS)) to each well, followed by incubation at 37 °C for 3 h in the dark. After, the plates were centrifuged at 2000 rpm for 15 min. The supernatant was removed, 200 μL dimethylsulfoxide (DMSO) was added to each well, and the plates were incubated for 30 min at 37 °C in the dark. Finally, the plates were read at 540 nm on a scanning multi-well spectrophotometer (Cary50MPR, Varian, Santa Clara, CA, USA) [53].

#### 3.2.3. NBT Assay

Since that during aging, among the ROS there are radicals as superoxide anion that can levels increase, [32] we used NBT assay in order to evaluate the antioxidant capacity of compound **6**. C2C12 cells (10^6^ cells) were stimulated with or w/o the compound **6** at 1 and 10 μM together with the RSV for 24 h, harvested, centrifuged for 5 min at 1200 rpm and resuspended in 1 mL 0.9% NaCl in which NBT (Nitro blue tetrazolium chloride) is dissolved 1 mg/mL. The cells were incubated for 3 h at 37 °C, centrifuged (for 10 min at 1200 rpm), resuspended in 1 mL DMSO and left 20’ in 37 °C. For the assay, the cells were plated in a 96 well plate (2 × 10^5^ cell/well) and read by spectrophotometer at 550 nm on a scanning multi-well spectrophotometer (Cary50MPR, Varian, Santa Clara, CA, USA).

#### 3.2.4. Measurement of Intracellular ROS

Intracellular ROS were quantified by the 2′,7′-dichlorodihydrofluorescein diacetate (H_2_DCF-DA Cat. No. D6883; Sigma). C2C12 cells were plated (2000 cells/well) into special optics 96-well plates (Corning-Costar, New York, NY, USA) and the cells were treated with RSV at 1 µM and Compound 6 at 1 and 10 µM for 24 h. After the incubation, cells were washed with an imaging buffer and treated with 300 µM H_2_O_2_ for an immediate fluorescence measurement. Plates were read every 50 s from 0 to 10 min for kinetic data analysis using a microplate reader (Synergy H1, BioTek), with excitation and emission wavelengths at 490 nm and 520 nm, respectively, and analyzed by Gen 5 version 2.08 (BioTek) [56].

#### 3.2.5. Statistical Analysis

The results of biological analysis are presented as the mean ± SEM of 3 different experiments. The statistical significance among multiple samples was determined with the one-way ANOVA analysis of variance with Bonferroni’s multiple comparison tests. When two groups were compared a non-parametric *t*-test was used comparing treated cells respect to the control. All statistical tests were performed via GraphPad Prism Software, version 7 (GraphPad Software, La Jolla, CA, USA).

### 3.3. Log P Studies

All chemical standards were synthesized in our laboratory as reported above. RSV (>99% purity grade) was obtained from Sigma-Aldrich (Milan, Italy). Methanol (HPLC-grade) was purchased from Honeywell (Morris Plains, NJ, USA). Deionized water (18.2 MΩ-cm at 25 °C) was generated by a Millipore Milli-Q Plus water (Millipore Bedford Corp., Bedford, MA, USA).

#### 3.3.1. Sample Preparation

Stock solutions of each compound were prepared as follow: 1 mg of the synthesized compound and RSV (control) was weighted and dissolved in 1 mL of dimethyl sulfoxide (DMSO). The working solutions were prepared in methanol at 100 µg/mL. 20 µL of working solutions was injected in HPLC system without pre-treatment.

#### 3.3.2. Chromatographic Conditions

The HPLC analyses for all new compounds were carried out in isocratic conditions with Milli-Q Water (18.2 MΩ-cm at 25 °C) as solvent A and MeOH as solvent B in different percentages reported in Table 5. RP-C18 stationary phase (LiChrosorb C_18_, 150 × 4.6 mm, 5 µm) column was used. All compounds were detected at their maximum wavelength: 322 nm for **1**, 301 nm for **2**, 324 nm for **3**, 324 nm for **4**, 373 nm for **5**, 322 nm for **6**, 320 nm for **7**, whereas RSV was detected at 306 nm. The total run time was kept until sample elution.

## 4. Conclusions

Emerging in vitro and preclinical data indicate that stilbene-containing compounds are capable of suppressing oxidative stress, but most of the experimental data has concentrated on RSV and only limited research has been carried out with RSV derivatives. This study aimed to explore as modification on the two aromatic rings of RSV influences the biological activity.

In summary, we designed and synthesized a series of novel RSV derivatives by introducing on 4′-OH stilbene moiety, chlorine atoms in 3′ and/or 2 and 4 positions. In 4 position, nitro or 3-fluoromethyl groups were introduced. All compounds were evaluated for their ability to modulate the cell vitality on C2C12 cell line. Among them, compound **6** was selected since it does not show cytotoxic effects, indeed seems to promote the proliferative capacity of the cells, also at 48 h. Moreover, compound **6** shows a remarkable antioxidant capacity and significantly reduces superoxide anion levels. The results obtained make us hypothesize that compound **6** interferes positively with the proliferative capacity of the cells and this could happen through its antioxidant capacity. Therefore, the present study demonstrates that substituted *trans-*4-hydroxy stilbenes represent promising compounds for the development of potential drugs that target oxidative stress in aging-related diseases. In particular, the derivative **6** could be considered as template for future investigation.

## Figures and Tables

**Table 1:** Retention times (t_R_) for **1**–**7** and RSV.

% MeOH	80	75	70	65	60	50	40	30	25	20
**1.**	2.230	3.007	4.485	7.441	13.529					
**2.**	2.615	3.412	5.353	9.004	15.779					
**3.**	4.006	6.433	11.254		39.949	173.677				
**4.**	5.719	9.004	17.887	36.844	76.574					
**5.**	2.368	3.091	4.821	7.906	13.950					
**6.**	3.075	4.956	8.689	16.674	35.018					
**7.**	2.430	3.578	5.667	10.032	19.349					
RSV						2.553	5.792	16.230	30.301	57.921

**Table 2:** Retention factor (*k*) for **1**–**7** and RSV.

% MeOH	80	75	70	65	60	50	40	30	25	20
**1.**	0.7490	1.3584	2.5176	4.8361	9.6110					
**2.**	1.0510	1.6761	3.1984	6.0620	11.3757					
**3.**	2.1420	4.0455	7.8267		30.3325	135.2173				
**4.**	3.4855	6.0620	13.0290	27.8973	59.0580					
**5.**	0.8573	1.4243	2.7812	5.2008	9.9412					
**6.**	1.4118	2.8871	5.8149	12.0776	26.4651					
**7.**	0.9059	1.8063	3.4447	6.8682	14.1757					
RSV						1.0024	3.5427	11.7294	22.7655	44.4282

**Table 3:** Log k for **1**–**7** and RSV.

% MeOH	80	75	70	65	60	50	40	30	25	20
**1.**	−0.1255	0.1330	0.4010	0.6845	0.9828					
**2.**	0.0216	0.2243	0.5049	0.7826	1.0560					
**3.**	0.3308	0.6070	0.8936		1.4819	2.1310				
**4.**	0.5423	0.7826	1.1149	1.4456	1.7713					
**5.**	−0.0669	0.1536	0.4442	0.7161	0.9974					
**6.**	0.1498	0.4605	0.7645	1.0820	1.4227					
**7.**	−0.0429	0.2568	0.5372	0.8368	1.1515					
RSV						0.0010	0.5493	1.0693	1.3573	1.6477

**Table 4:** Experimental and theoretical *Log P* of **1**–**7** and RSV.

Compound	*Log P* * ± SD	*Log P* #
**1.**	4.29 ± 0.07	4.88
**2.**	4.20 ± 0.12	4.85
**3.**	5.11 ± 0.07	5.59
**4.**	5.50 ± 0.14	6.27
**5.**	4.22 ± 0.09	4.59
**6.**	5.21 ± 0.06	5.73
**7.**	4.70 ± 0.05	5.05
RSV	2.47 ± 0.11	2.83

* *Log P* estimated by RP-HPLC; # *Log P* calculated using ChemDraw; SD = Standard Deviation evaluated at 95% confidence interval.

**Table 5:** Different tested mobile phase percentages.

FLOW RATE (mL/min)	% A H_2_O Milli-Q	% B Methanol
1	20	80
1	25	75
1	30	70
1	35	65
1	40	60
1	50	50
1	60	40
1	70	30
1	75	25
1	80	20

## Supplementary Materials

The following are available online, Table S1. Different tested mobile phase percentages, Figure S1. Chromatogram of RSV, Figure S2. Chromatogram of compound **1**, Figure S3. Chromatogram of compound **2**, Figure S4. Chromatogram of compound **3**, Figure S5. Chromatogram of compound **4**, Figure S6. Chromatogram of compound **5**, Figure S7. Chromatogram of compound **6**, Figure S8. Chromatogram of compound **7**.

## Abbreviations

Resveratrol	RSV
structure-activity relationship	SAR
type 2 diabetes	T2D
age-related macular degeneration	AMD
Alzheimer’s disease	AD
Parkinson’s disease	PD
reactive oxygen species	ROS
glucose restriction	GR
xanthine oxidase	XO
