# Supplementary material for: Synthesis and Biological Evaluation of Halogenated E-Stilbenols as Promising Antiaging Agents
Source: Molecules. 2020 Dec 7;25(23):5770. doi: 10.3390/molecules25235770 (PMC7731283; doi:10.3390/molecules25235770)
Supplement: Supplementary file 1 [file molecules-25-05770-s001.pdf]

Supporting Information

Article

Synthesis and Biological Evaluation of Halogenated *E*-Stilbenols as Promising Antiaging Agents

Ester Sara Di Filippo [1,†], Letizia Giampietro [2,†], Barbara De Filippis [2,*], Marwa Balaha [2,3], Vincenzo Ferrone [2], Marcello Locatelli [2], Tiziana Pietrangelo [1], Angela Tartaglia [2], Rosa Amoroso [2] and Stefania Fulle [1]

1 Department of Neuroscience Imaging and Clinical Sciences, Interuniversity Institute of Myology, University "G. d'Annunzio" of Chieti-Pescara, 66100 Chieti, Italy; es.difilippo@unich.it (E.S.D.F.); tiziana.pietrangelo@unich.it (T.P.); sfulle@unich.it (S.F.)
2 Department of Pharmacy, University "G. d'Annunzio" of Chieti-Pescara, 66100 Chieti, Italy; letizia.giampietro@unich.it (L.G.); marwa.balaha@pharm.tanta.edu.eg (M.B.); Vincenzo.ferrone@unich.it (V.F.); marcello.locatelli@unich.it (M.L.); angela.tartaglia@unich.it (A.T.); rosa.amoroso@unich.it (R.A.)
3 Department of Pharmaceutical Chemistry, Faculty of Pharmacy, Kafrelsheikh University, Kafr El Sheikh 33516, Egypt
* Correspondence: barbara.defilippis@unich.it; Tel.: +39-0871-3554685
† These authors contributed equally to this work

Log P studies

All chemical standards were synthesized in our laboratory as reported above. RSV (>99% purity grade) was obtained from Sigma-Aldrich (Milan, Italy). Methanol (HPLC-grade) was purchased from Honeywell (New Jersey, USA). Deionized water (18.2MΩ-cm at 25 °C) was generated by a Millipore Milli-Q Plus water (Millipore Bedford Corp., Bedford, MA, USA).

Sample preparation

Stock solutions of each compound were prepared as follow: 1 mg of the synthesized compound and RSV (control) was weighted and dissolved in 1 mL of dimethyl sulfoxide (DMSO). The working solutions were prepared in methanol at100 µg/mL. 20 µL of working solutions was injected in HPLC system without pre-treatment.

Chromatographic conditions

The HPLC analyses for all new compounds were carried out in isocratic conditions with Milli-Q Water (18.2 MΩ-cm at 25 °C) as solvent A and MeOH as solvent B in different percentages reported in **Table 5**. RP-C18 stationary phase (LiChrosorb C_{18}, 150x4.6 mm, 5µm) column was used. All compounds were detected at their maximum wavelength: 322 nm for **1**, 301 nm for **2**, 324 nm for **3**, 324 nm for **4**, 373 nm for **5**, 322 nm for **6**, 320 nm for **7**, whereas RSV was detected at 306 nm. The total run time was kept until sample elution.

Table 1. Different tested mobile phase percentages.

FLOW RATE (mL/min)	% A H_2O Milli-Q	% B Methanol
1	20	80
1	25	75
1	30	70
1	35	65
1	40	60
1	50	50
1	60	40
1	70	30
1	75	25
1	80	20

All chromatograms detected at the maximum wavelength, the peak area and the purity (%) of new synthesized compounds are showed in **Figures S1** to **S8**.

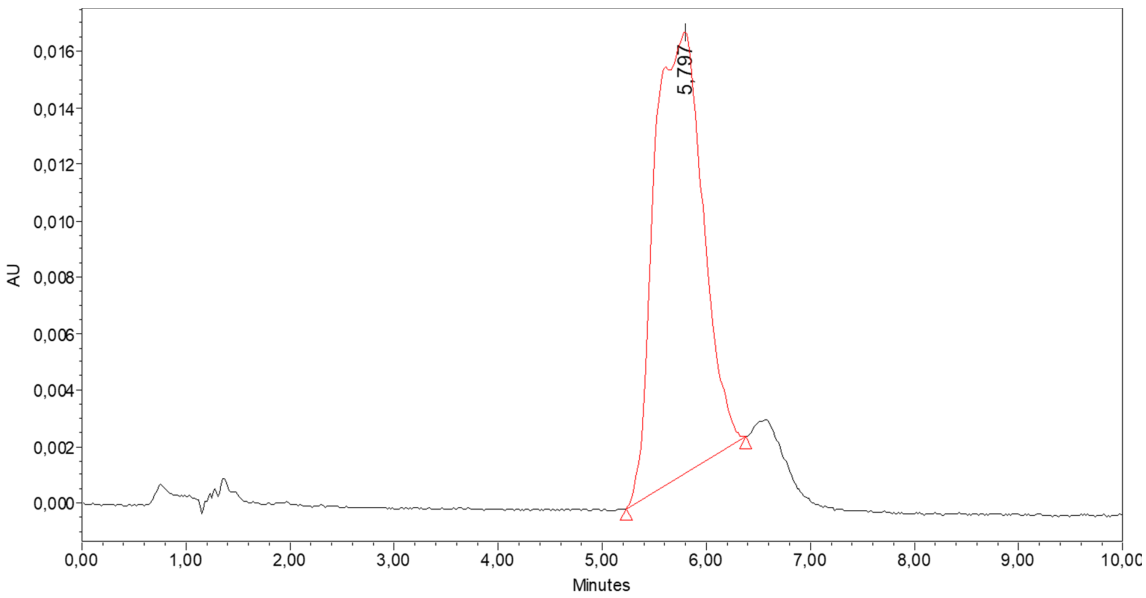

% H_2O	% MeOH	Area	λ (nm)	Purity (%)
60	40	513348	306	94.24

Figure 1. Chromatogram of RSV.

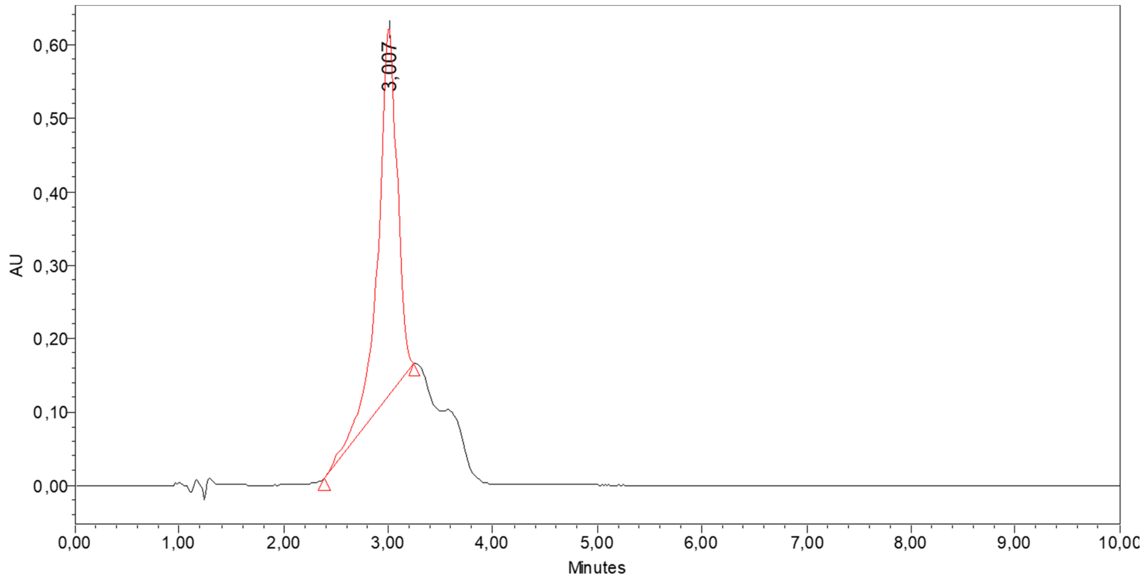

% H_2O	% MeOH	Area	λ (nm)	Purity (%)
25	75	6430377	322	85.47

Figure 2. Chromatogram of compound **1**.

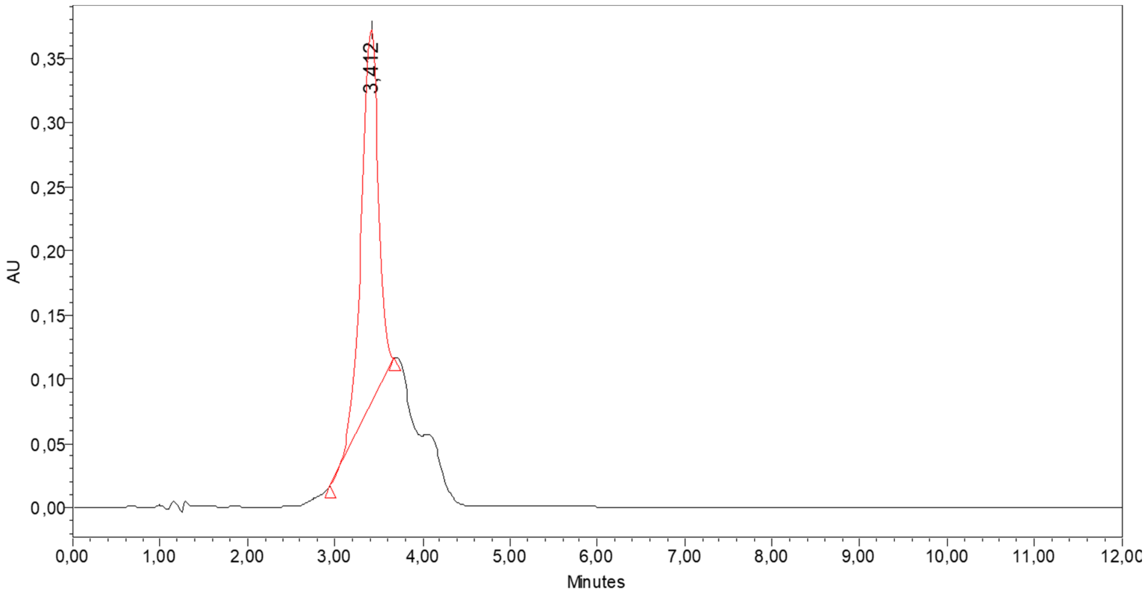

% H_2O	% MeOH	Area	λ (nm)	Purity (%)
25	75	3981565	301	88.32

Figure 3. Chromatogram of compound **2**.

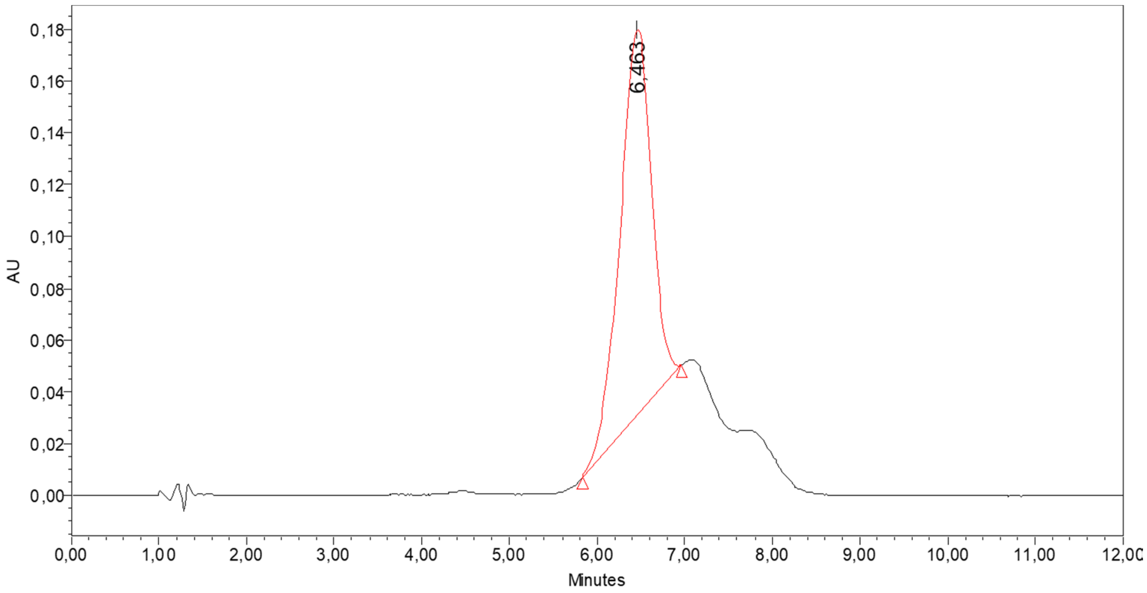

% H_2O	% MeOH	Area	λ (nm)	Purity (%)
25	75	3141407	324	69.40

Figure 4. Chromatogram of compound **3**.

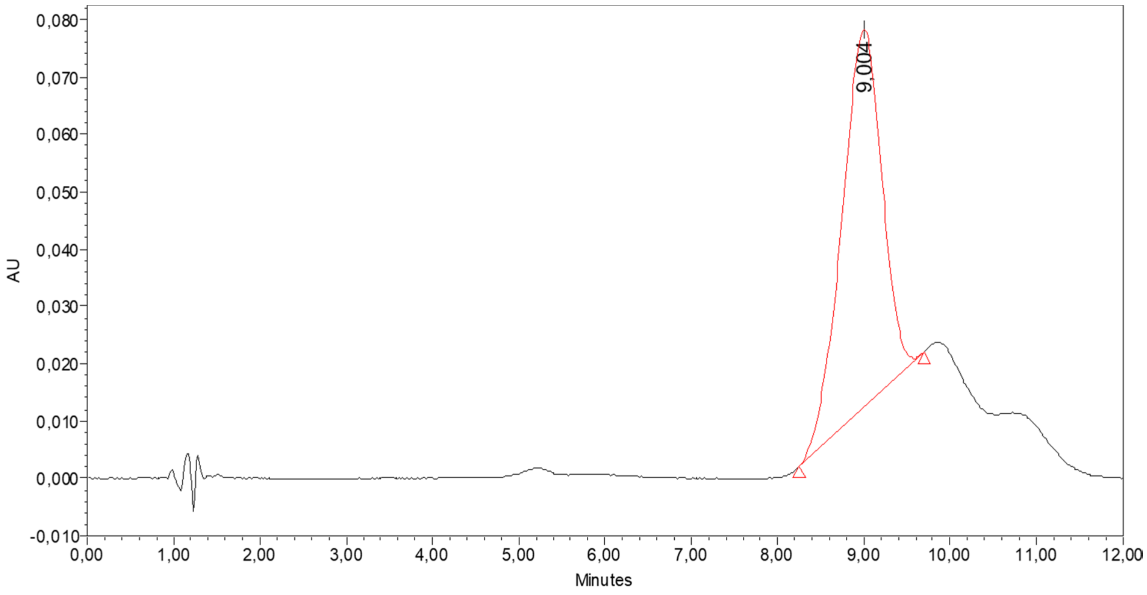

% H_2O	% MeOH	Area	λ (nm)	Purity (%)
25	75	2129280	324	79.91

Figure 5. Chromatogram of compound **4**.

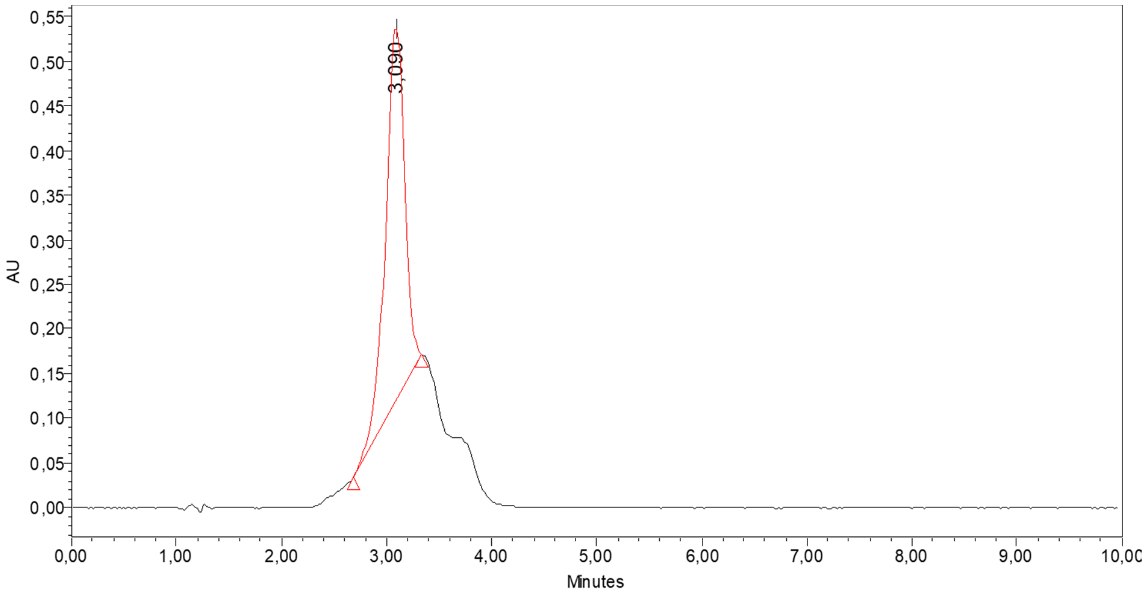

% H_2O.	% MeOH	Area	λ (nm)	Purity (%)
25	75	5232835	373	93.31

Figure 6. Chromatogram of compound **5**.

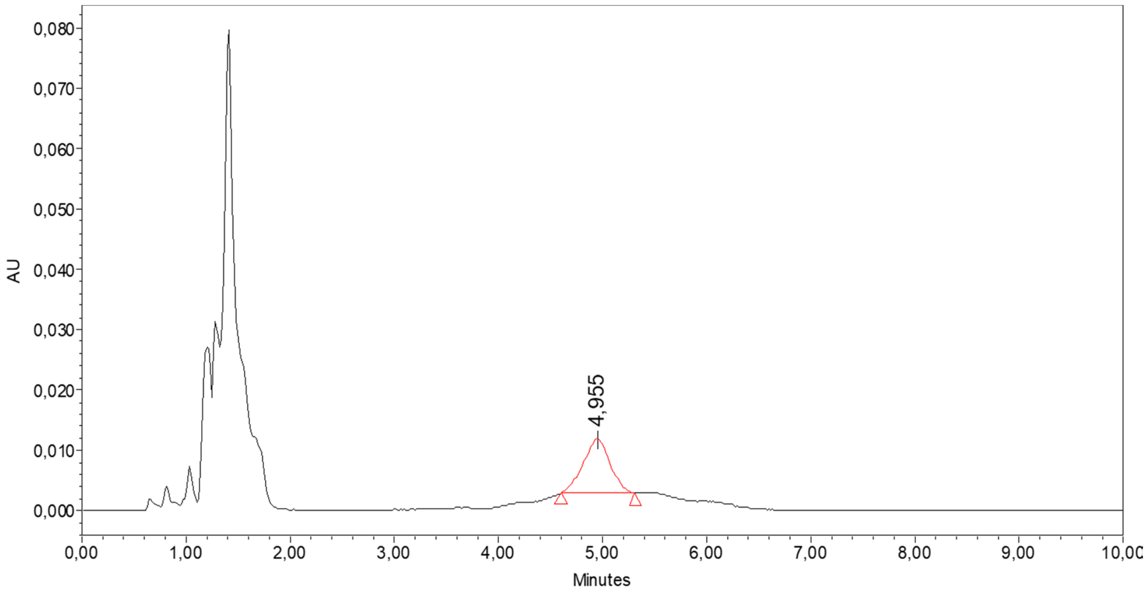

% H_2O	% MeOH	Area	λ (nm)	Purity (%)
25	75	158468	322	15.25

Figure 7. Chromatogram of compound **6**.

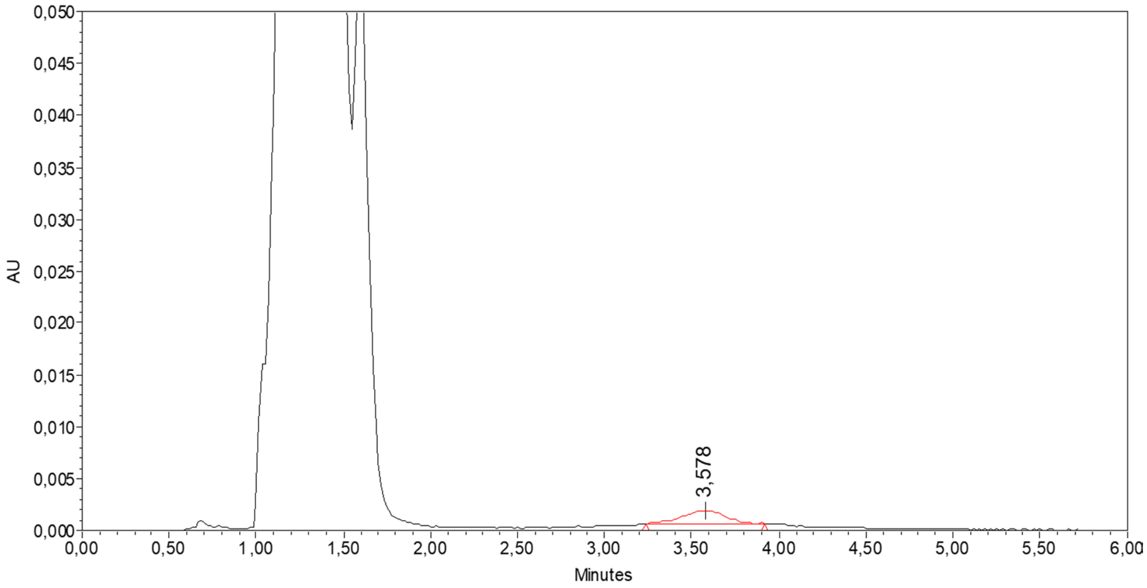

% H_2O	% MeOH	Area	λ (nm)	Purity (%)
25	75	21044	320	0.73

Figure 8. Chromatogram of compound **7**.

Compound 1

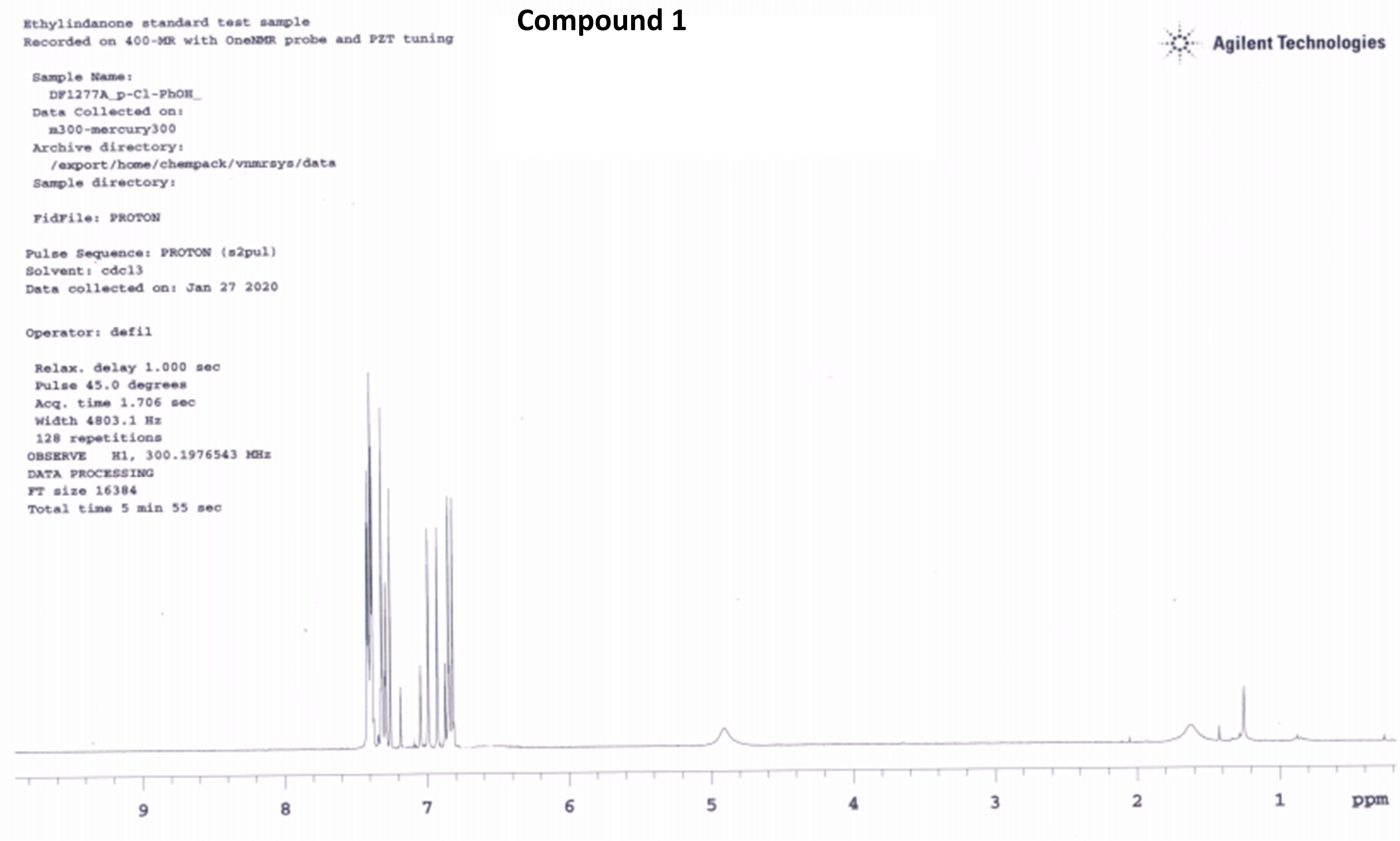
Ethylindanone standard test sample
Recorded on 400-MR with OneNMR probe and PZT tuning
Agilent Technologies
Sample Name:
DF1277A_p-Cl-PhOH_
Data Collected on:
m300-mercury300
Archive directory:
/export/home/chempack/vnmrsys/data
Sample directory:
FidFile: PROTON
Pulse Sequence: PROTON (s2pul)
Solvent: cdcl3
Data collected on: Jan 27 2020
Operator: defil
Relax. delay 1.000 sec
Pulse 45.0 degrees
Acq. time 1.706 sec
Width 4803.1 Hz
128 repetitions
OBSERVE H1, 300.1976543 MHz
DATA PROCESSING
FT size 16384
Total time 5 min 55 sec
9
8
7
6
5
4
3
2
1
ppm

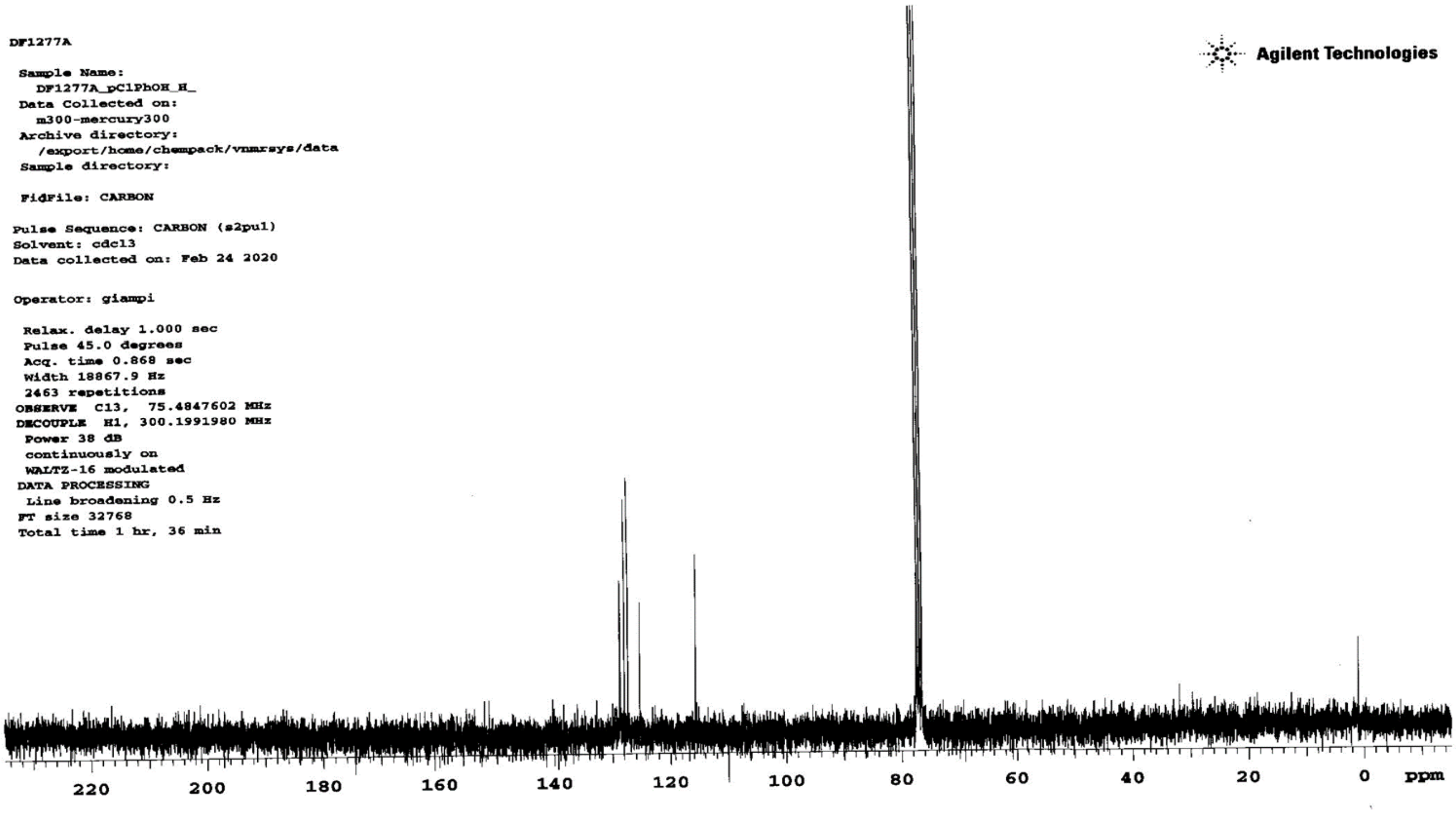
DF1277A
Sample Name:
DF1277A_pClPhOH_H_
Data Collected on:
m300-mercury300
Archive directory:
/export/home/chempack/vnmrsys/data
Sample directory:
FidFile: CARBON
Pulse Sequence: CARBON (s2pul)
Solvent: cdcl3
Data collected on: Feb 24 2020
Operator: giampi
Relax. delay 1.000 sec
Pulse 45.0 degrees
Acq. time 0.868 sec
Width 18867.9 Hz
2463 repetitions
OBSERVE C13, 75.4847602 MHz
DECOUPLE H1, 300.1991980 MHz
Power 38 dB
continuously on
WALTZ-16 modulated
DATA PROCESSING
Line broadening 0.5 Hz
FT size 32768
Total time 1 hr, 36 min
Agilent Technologies
220 200 180 160 140 120 100 80 60 40 20 0 ppm

Compound 2

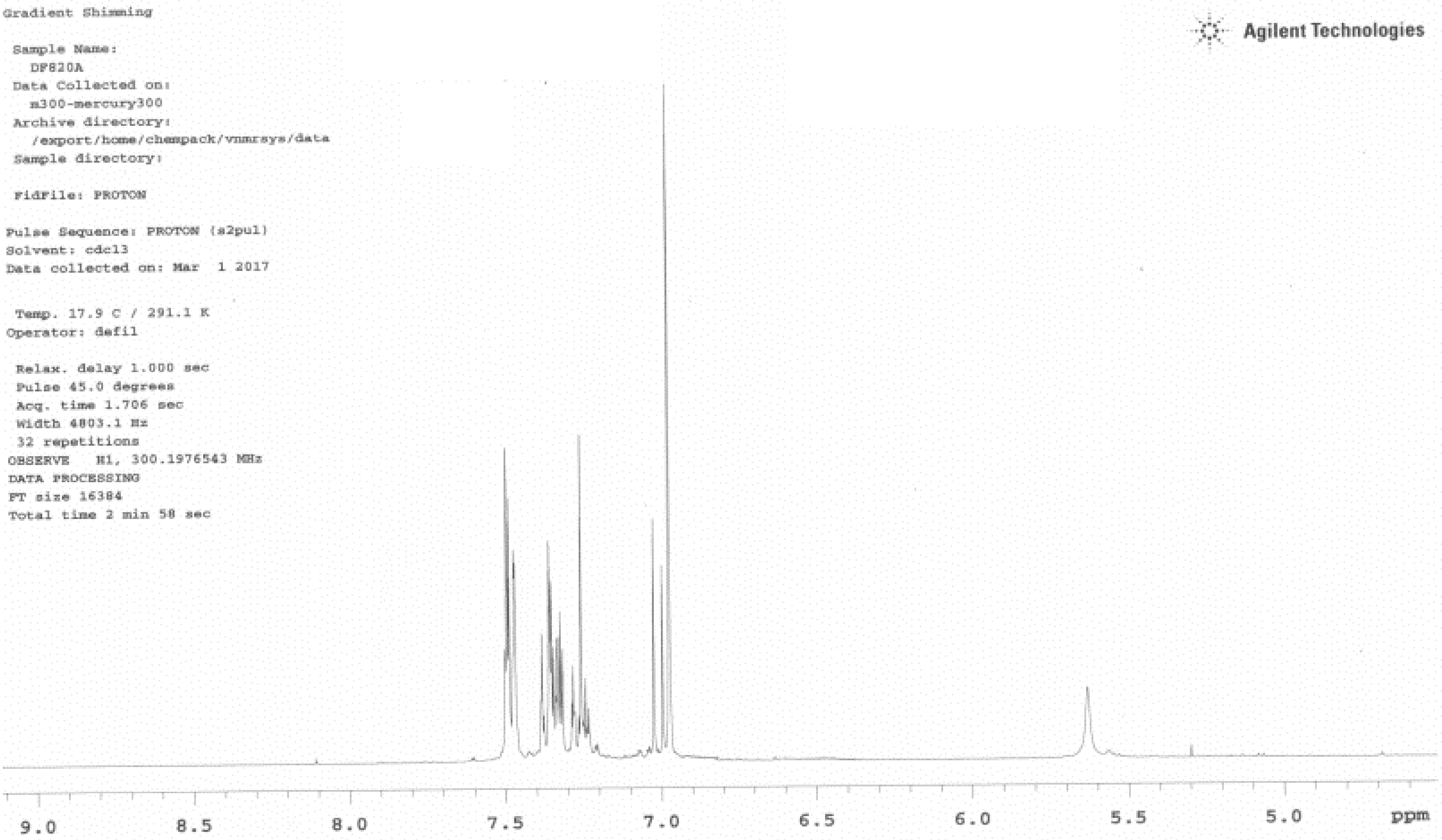
Gradient Shimming
Sample Name:
DF820A
Data Collected on:
m300-mercury300
Archive directory:
/export/home/chempack/vnmrsys/data
Sample directory:
FidFile: PROTON
Pulse Sequence: PROTON (s2pul)
Solvent: cdcl3
Data collected on: Mar 1 2017
Temp. 17.9 C / 291.1 K
Operator: defil
Relax. delay 1.000 sec
Pulse 45.0 degrees
Acq. time 1.706 sec
Width 4803.1 Hz
32 repetitions
OBSERVE H1, 300.1976543 MHz
DATA PROCESSING
FT size 16384
Total time 2 min 58 sec
Agilent Technologies
9.0
8.5
8.0
7.5
7.0
6.5
6.0
5.5
5.0
ppm

Sample Name:
DF820A
Data Collected on:
m300-mercury300
Archive directory:
/export/home/chempack/vnmrs[illegible]
Sample directory:

FidFile: CARBON

Pulse Sequence: CARBON (s2pul)
Solvent: cdcl3
Data collected on: Mar 1 2017

Temp. 25.0 C / 298.1 K
Operator: defil

Relax. delay 1.000 sec
Pulse 45.0 degrees
Acq. time 0.868 sec
Width 18867.9 Hz
500 repetitions
OBSERVE C13, 75.4847602 MHz
DECOUPLE H1, 300.1991980 MHz
Power 38 dB
continuously on
WALTZ-16 modulated
DATA PROCESSING
Line broadening 0.5 Hz
FT size 32768
Total time 16 min

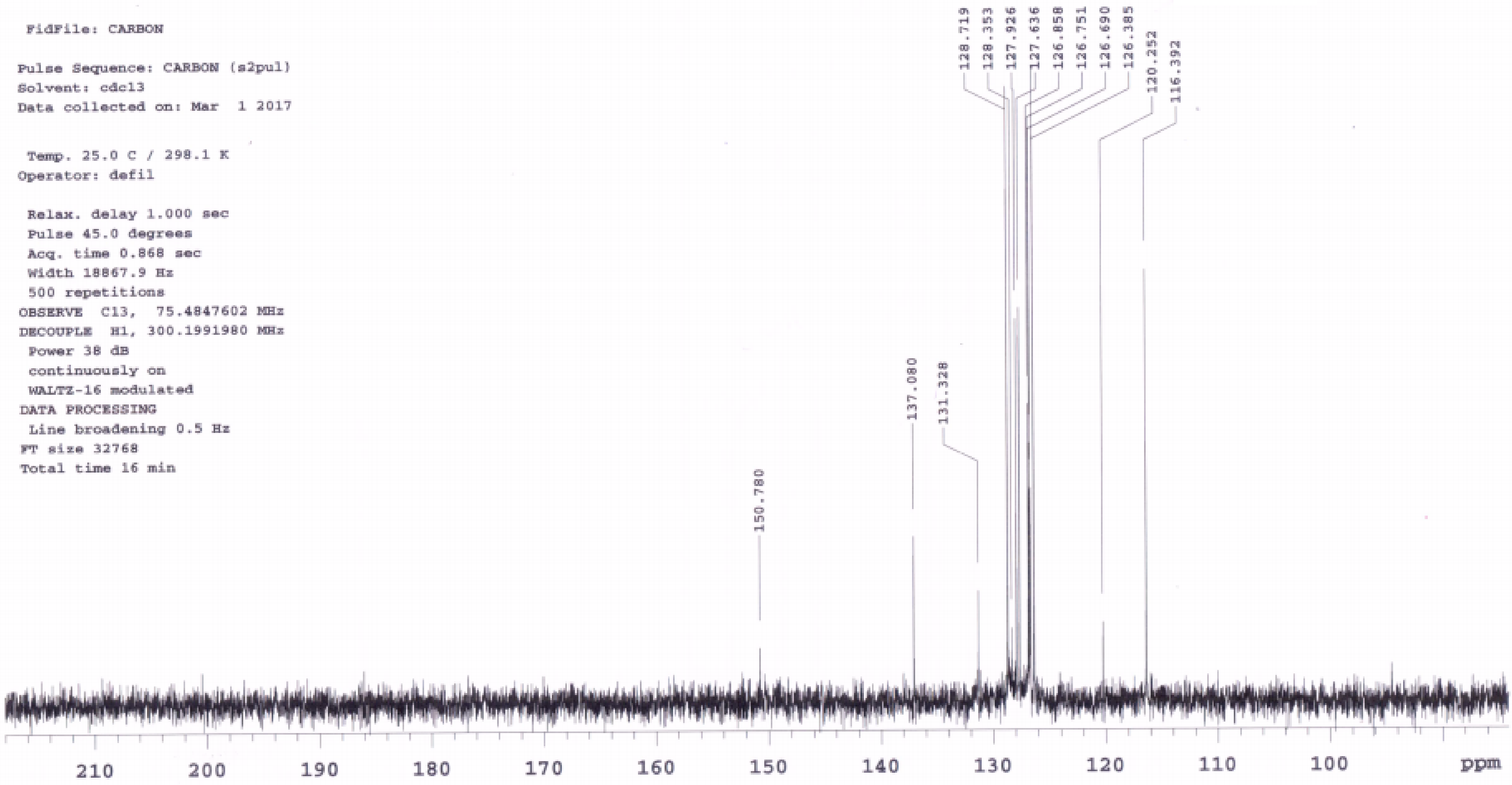

Compound 3

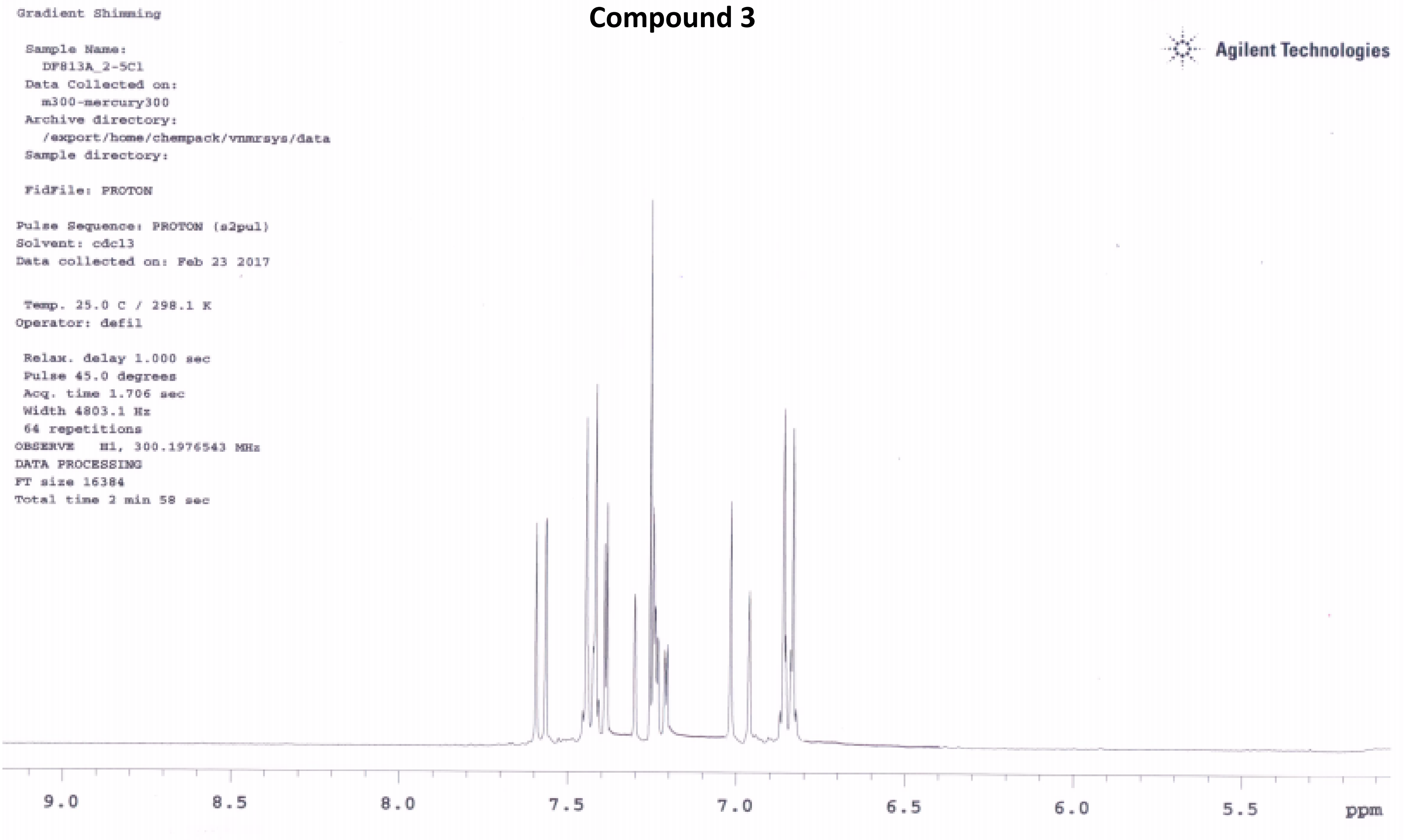
Gradient Shimming
Sample Name:
DF813A_2-5Cl
Data Collected on:
m300-mercury300
Archive directory:
/export/home/chempack/vnmrsys/data
Sample directory:
FidFile: PROTON
Pulse Sequence: PROTON (s2pul)
Solvent: cdcl3
Data collected on: Feb 23 2017
Temp. 25.0 C / 298.1 K
Operator: defil
Relax. delay 1.000 sec
Pulse 45.0 degrees
Acq. time 1.706 sec
Width 4803.1 Hz
64 repetitions
OBSERVE H1, 300.1976543 MHz
DATA PROCESSING
FT size 16384
Total time 2 min 58 sec
Agilent Technologies
9.0
8.5
8.0
7.5
7.0
6.5
6.0
5.5
ppm

DF1279 A
saved in GL

Sample Name:
DF1279A_diClPhOH_H_
Data Collected on:
m300-mercury300
Archive directory:
/export/home/chempack/vnmrsys/data
Sample directory:

FidFile: CARBON

Pulse Sequence: CARBON (s2pul)
Solvent: cdcl3
Data collected on: Feb 12 2020

Operator: giampi

Relax. delay 1.000 sec
Pulse 45.0 degrees
Acq. time 0.868 sec
Width 18867.9 Hz
1400 repetitions
OBSERVE C13, 75.4847602 MHz
DECOUPLE H1, 300.1991980 MHz
Power 38 dB
continuously on
WALTZ-16 modulated
DATA PROCESSING
Line broadening 0.5 Hz
FT size 32768
Total time 45 min

Agilent Technologies

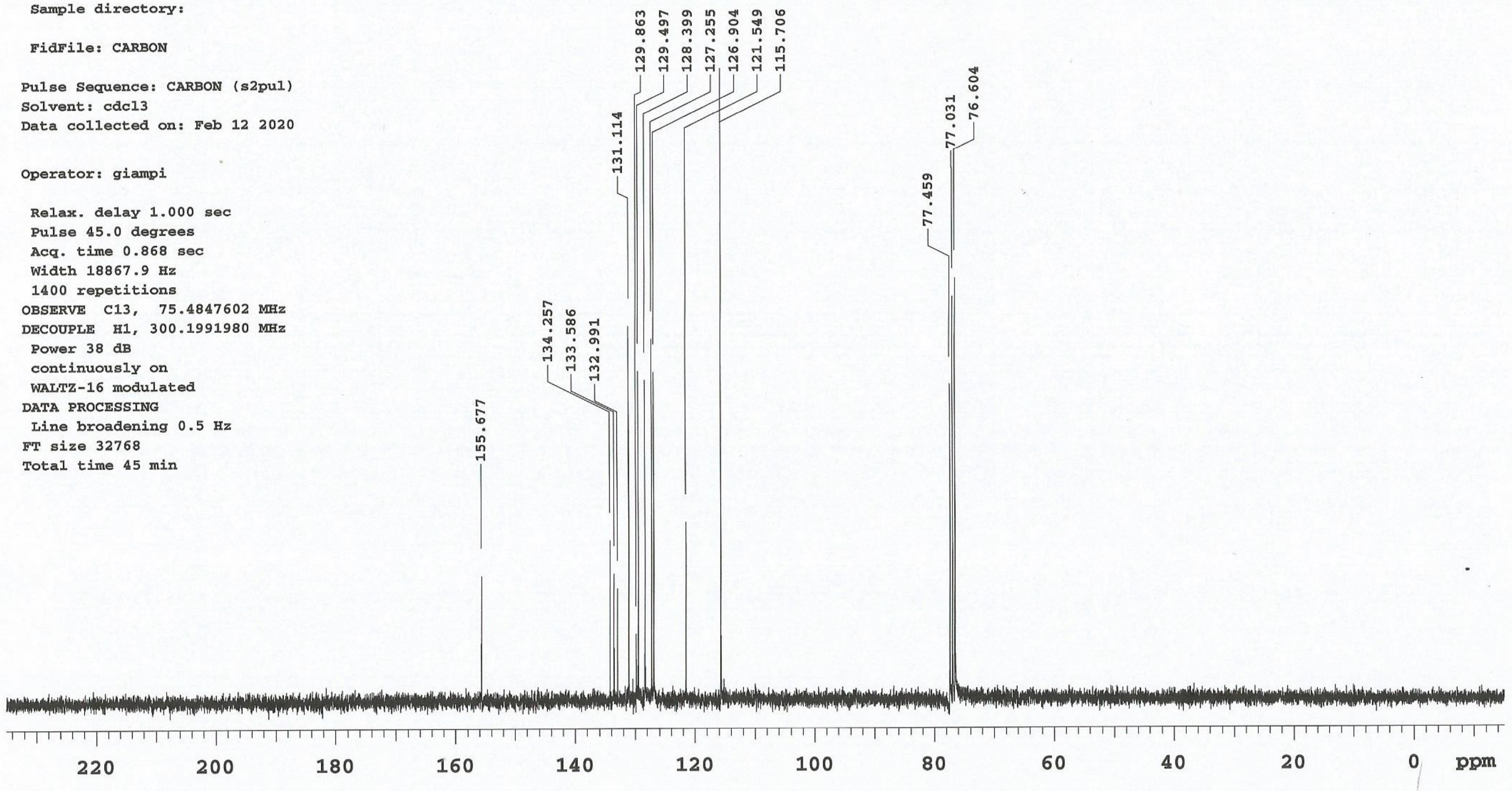

Compound 4

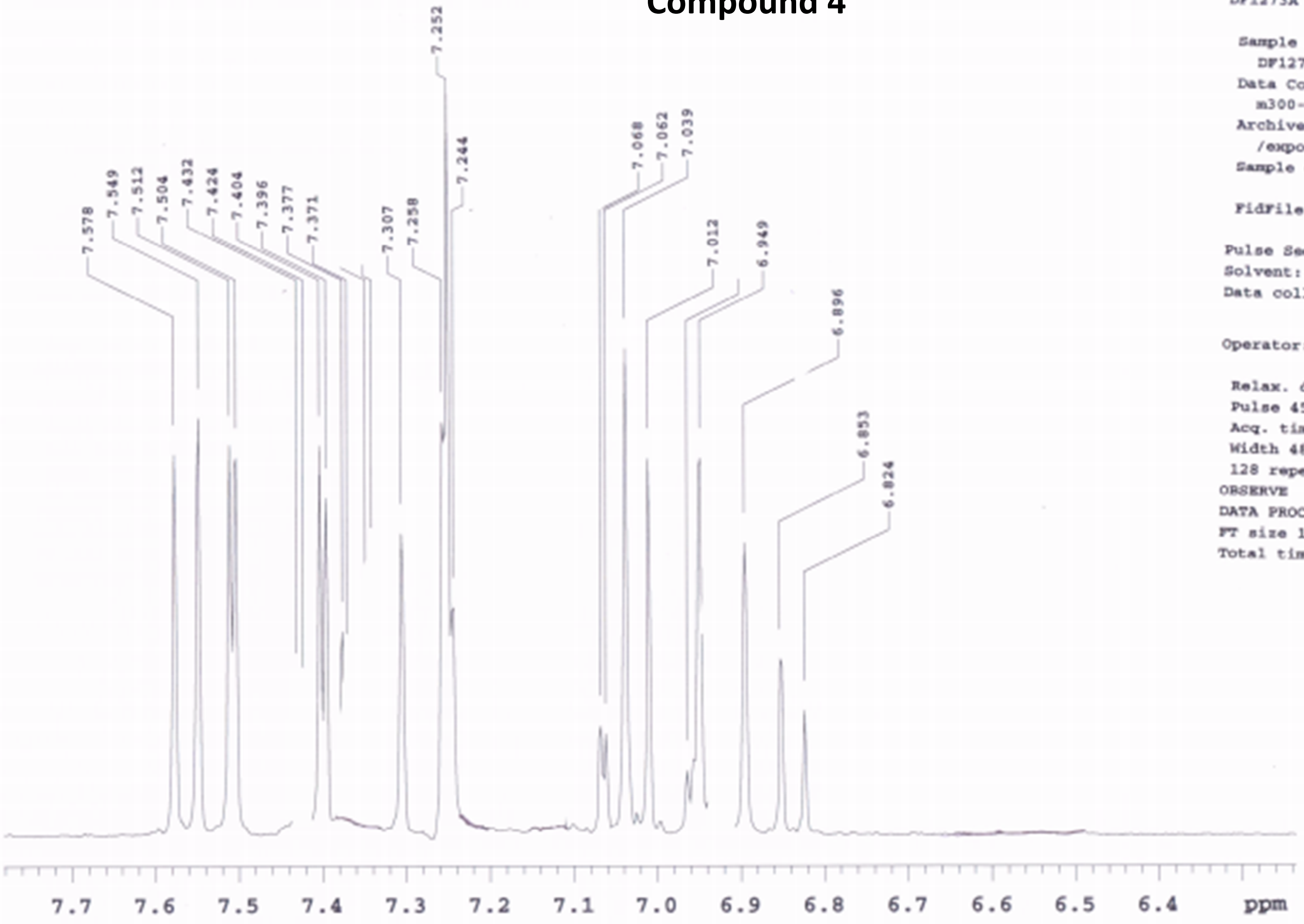

DF1273A 2 spettro

Sample Name:
DF1273A
Data Collected on:
m300-mercury300
Archive directory:
/export/home/chempack/vnmrsys/data
Sample directory:

FidFile: PROTON

Pulse Sequence: PROTON (s2pul)
Solvent: cdcl3
Data collected on: Feb 13 2020

Operator: giampi

Relax. delay 1.000 sec
Pulse 45.0 degrees
Acq. time 1.706 sec
Width 4803.1 Hz
128 repetitions
OBSERVE H1, 300.1976543 MHz
DATA PROCESSING
FT size 16384
Total time 5 min 55 sec

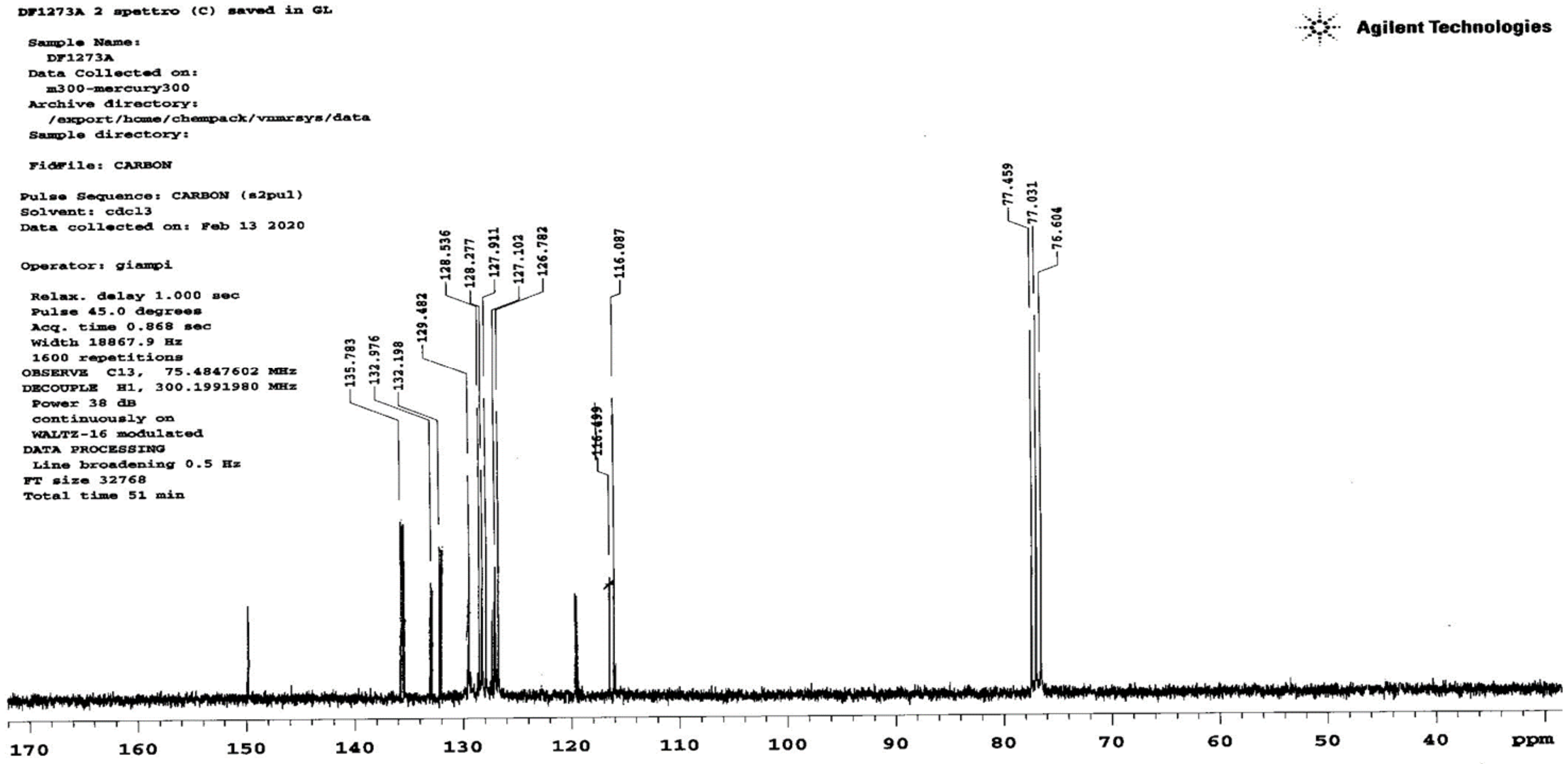
DF1273A 2 spettro (C) saved in GL
Sample Name:
DF1273A
Data Collected on:
m300-mercury300
Archive directory:
/export/home/chempack/vnmrsys/data
Sample directory:
FidFile: CARBON
Pulse Sequence: CARBON (s2pul)
Solvent: cdcl3
Data collected on: Feb 13 2020
Operator: giampi
Relax. delay 1.000 sec
Pulse 45.0 degrees
Acq. time 0.868 sec
Width 18867.9 Hz
1600 repetitions
OBSERVE C13, 75.4847602 MHz
DECOUPLE H1, 300.1991980 MHz
Power 38 dB
continuously on
WALTZ-16 modulated
DATA PROCESSING
Line broadening 0.5 Hz
FT size 32768
Total time 51 min
Agilent Technologies
135.783
132.976
132.198
129.482
128.536
128.277
127.911
127.102
126.782
116.499
116.087
77.459
77.031
76.604
170
160
150
140
130
120
110
100
90
80
70
60
50
40
ppm

Compound 5

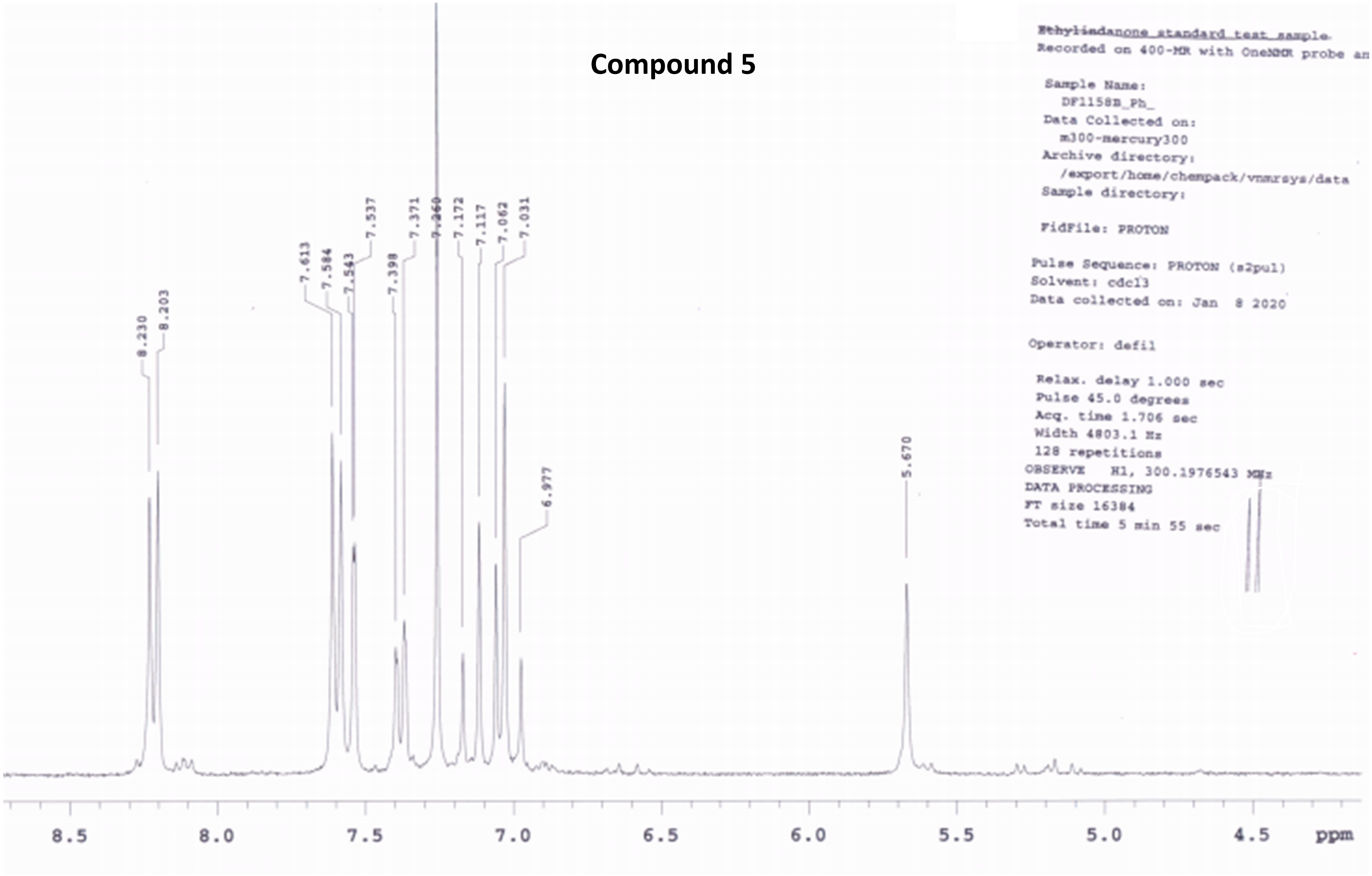
Ethylindanone standard test sample
Recorded on 400-MR with OneNMR probe an
Sample Name:
DF1158B_Ph_
Data Collected on:
m300-mercury300
Archive directory:
/export/home/chempack/vnmrsys/data
Sample directory:
FidFile: PROTON
Pulse Sequence: PROTON (s2pul)
Solvent: cdcl3
Data collected on: Jan 8 2020
Operator: defil
Relax. delay 1.000 sec
Pulse 45.0 degrees
Acq. time 1.706 sec
Width 4803.1 Hz
128 repetitions
OBSERVE H1, 300.1976543 MHz
DATA PROCESSING
FT size 16384
Total time 5 min 55 sec
8.230
8.203
7.613
7.584
7.543
7.537
7.398
7.371
7.260
7.172
7.117
7.062
7.031
6.977
5.670
8.5
8.0
7.5
7.0
6.5
6.0
5.5
5.0
4.5
ppm

Compound 6

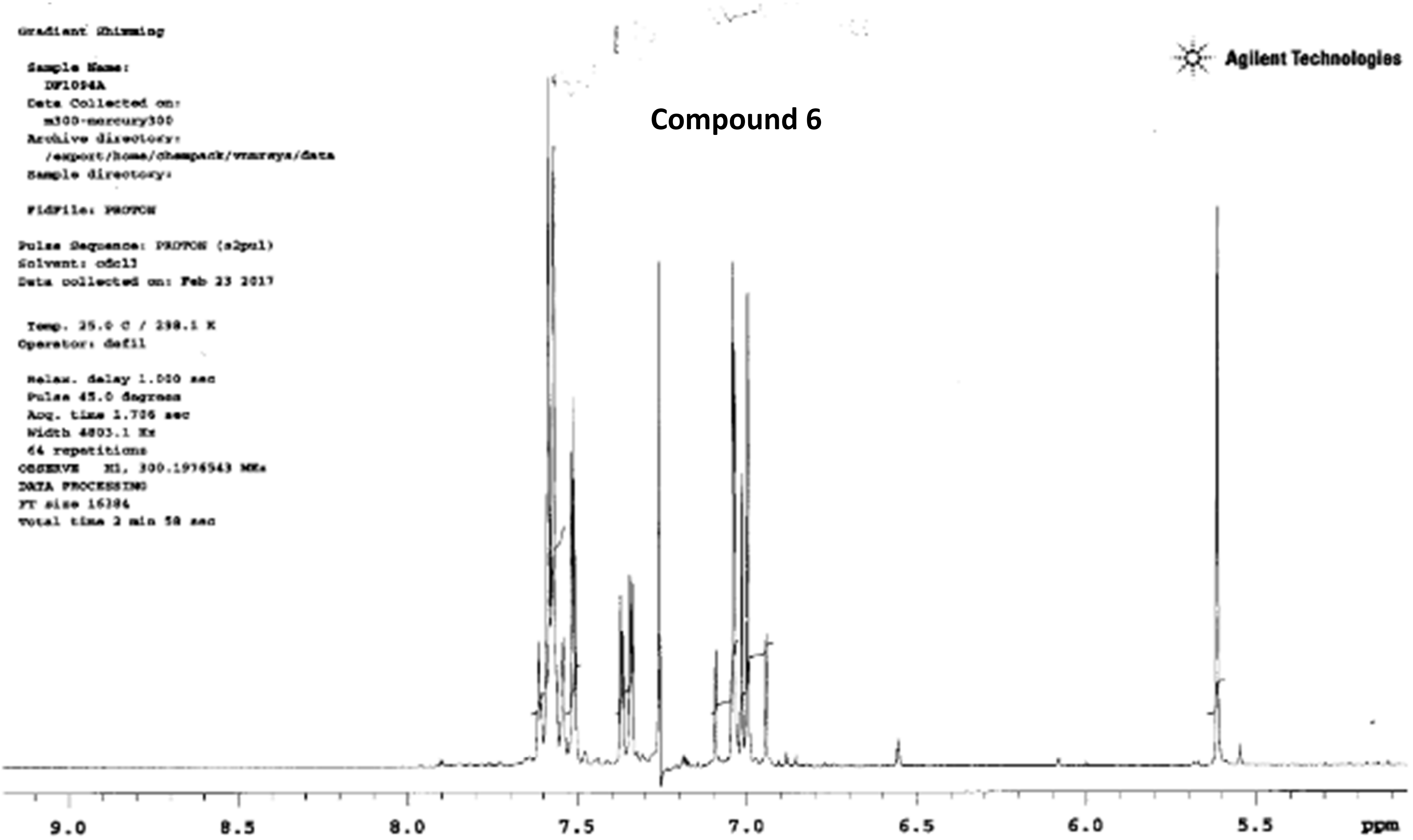
Gradient Shimming
Sample Name:
DF1094A
Data Collected on:
m300-mercury300
Archive directory:
/export/home/chempack/vnmrsys/data
Sample directory:
FidFile: PROTON
Pulse Sequence: PROTON (s2pul)
Solvent: cdcl3
Data collected on: Feb 23 2017
Temp. 25.0 C / 298.1 K
Operator: defil
Relax. delay 1.000 sec
Pulse 45.0 degrees
Acq. time 1.706 sec
Width 4803.1 Hz
64 repetitions
OBSERVE H1, 300.1976543 MHz
DATA PROCESSING
FT size 16384
Total time 2 min 58 sec
Agilent Technologies
9.0
8.5
8.0
7.5
7.0
6.5
6.0
5.5
ppm

DF 1094 A

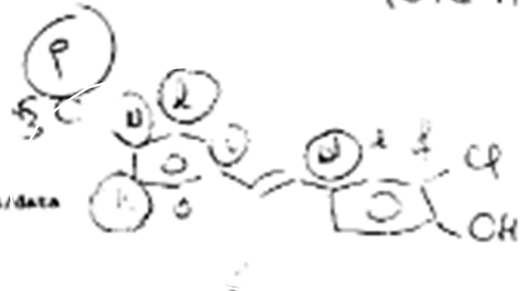

Gradient Shimming

Sample Name:
DF1094A
Data Collected on:
m300-mercury300
Archive directory:
/export/home/chempack/vnmrsys/data
Sample directory:

FidFile: CARBON

Pulse Sequence: CARBON (s2pul)
Solvent: cdcl3
Data collected on: Feb 27 2017

Temp. 25.0 C / 298.1 K
Operator: defil

Relax. delay 1.000 sec
Pulse 45.0 degrees
Acq. time 0.868 sec
Width 18867.9 Hz
600 repetitions
OBSERVE C13, 75.4847602 MHz
DECOUPLE H1, 300.1951980 MHz
Power 38 dB
continuously on
WALTZ-16 modulated
DATA PROCESSING
Line broadening 0.5 Hz
FT size 32768
Total time 19 min

Agilent Technologies

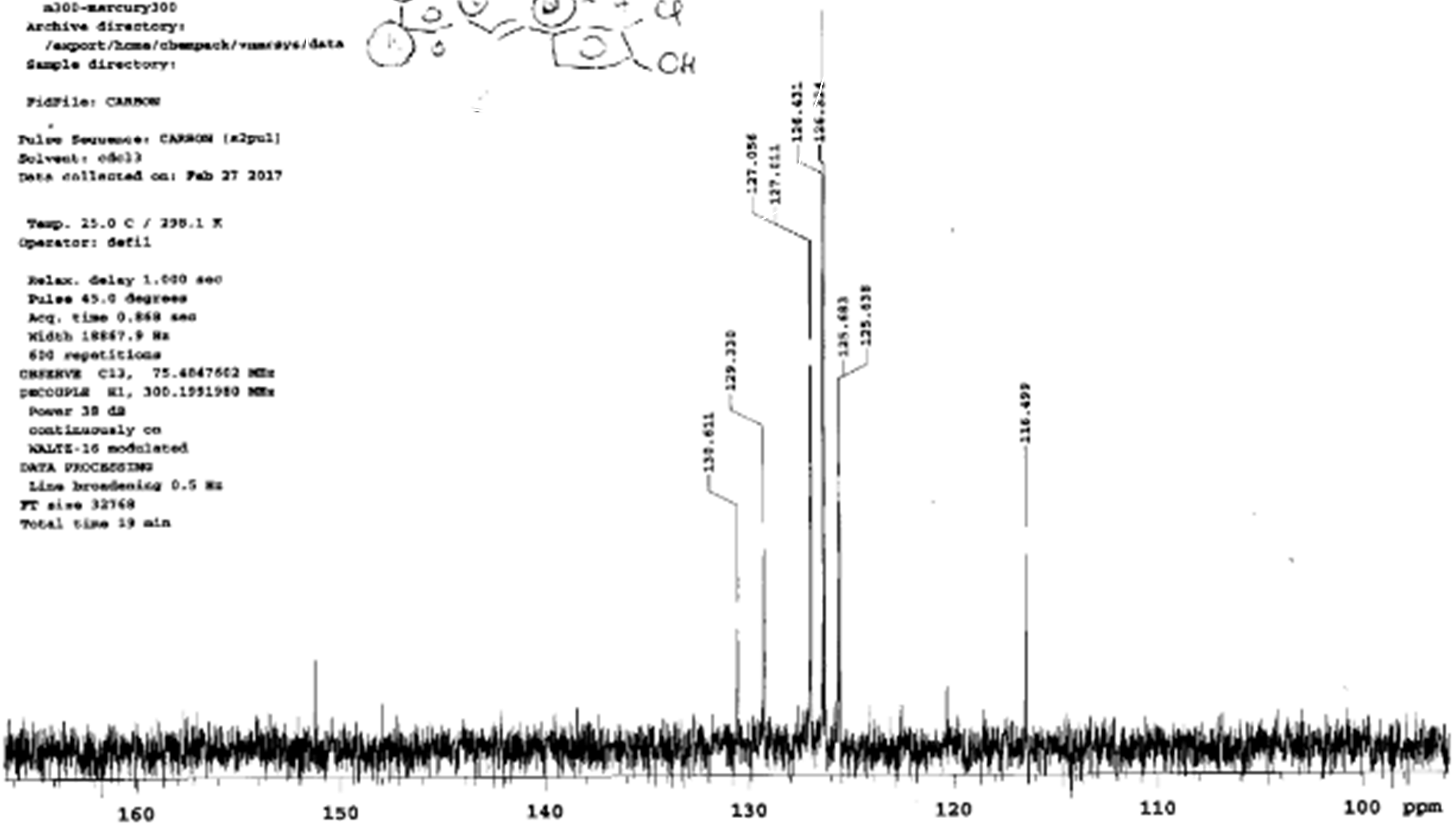

DF1278A

Sample Name:
DF1278A_CF3-SLB-_H_H_
Data Collected on:
m300-mercury300
Archive directory:
/export/home/chempack/vnmrsys/data
Sample directory:

FidFile: PROTON

Pulse Sequence: PROTON (s2pul)
Solvent: acetone
Data collected on: Mar 11 2020

Temp. 20.4 C / 293.6 K
Operator: giampi

Relax. delay 1.000 sec
Pulse 45.0 degrees
Acq. time 1.706 sec
Width 4803.1 Hz
64 repetitions
OBSERVE H1, 300.1992123 MHz
DATA PROCESSING
FT size 16384
Total time 5 min 55 sec

Compound 7

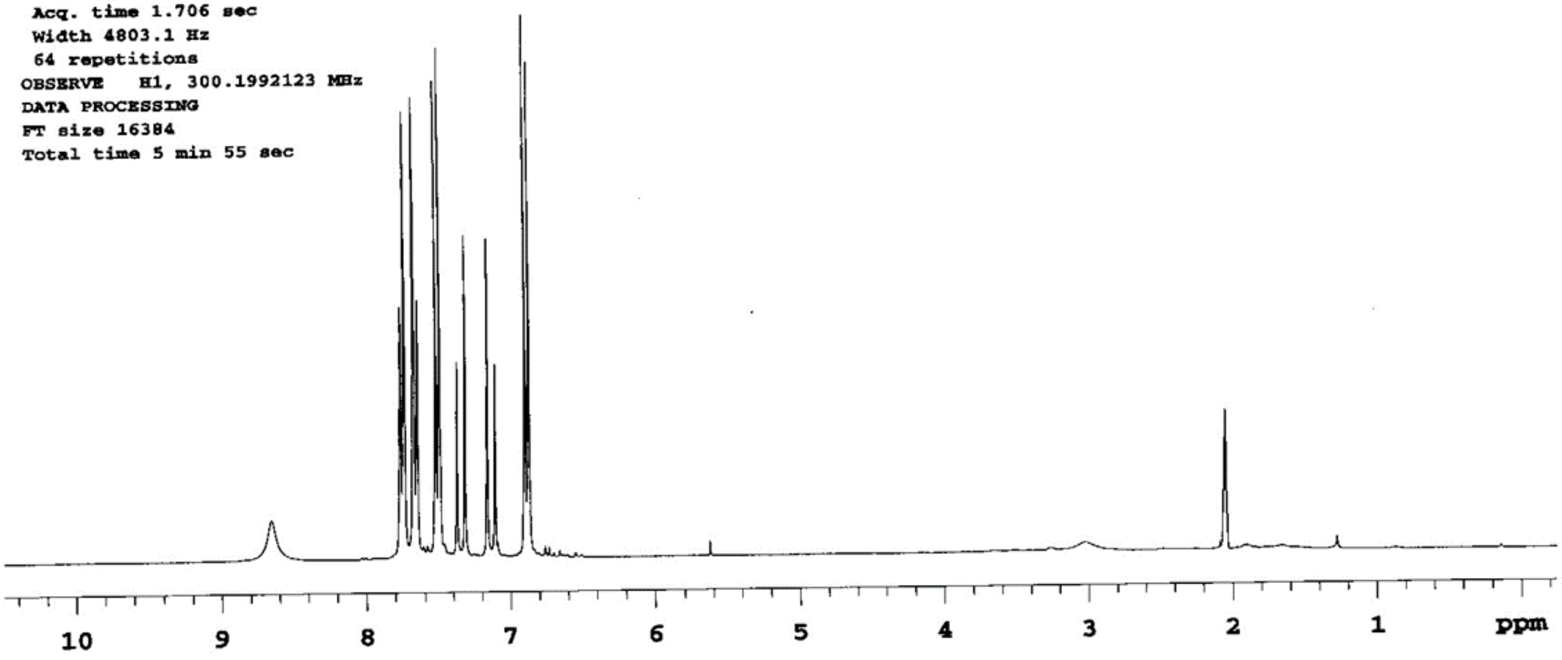

22

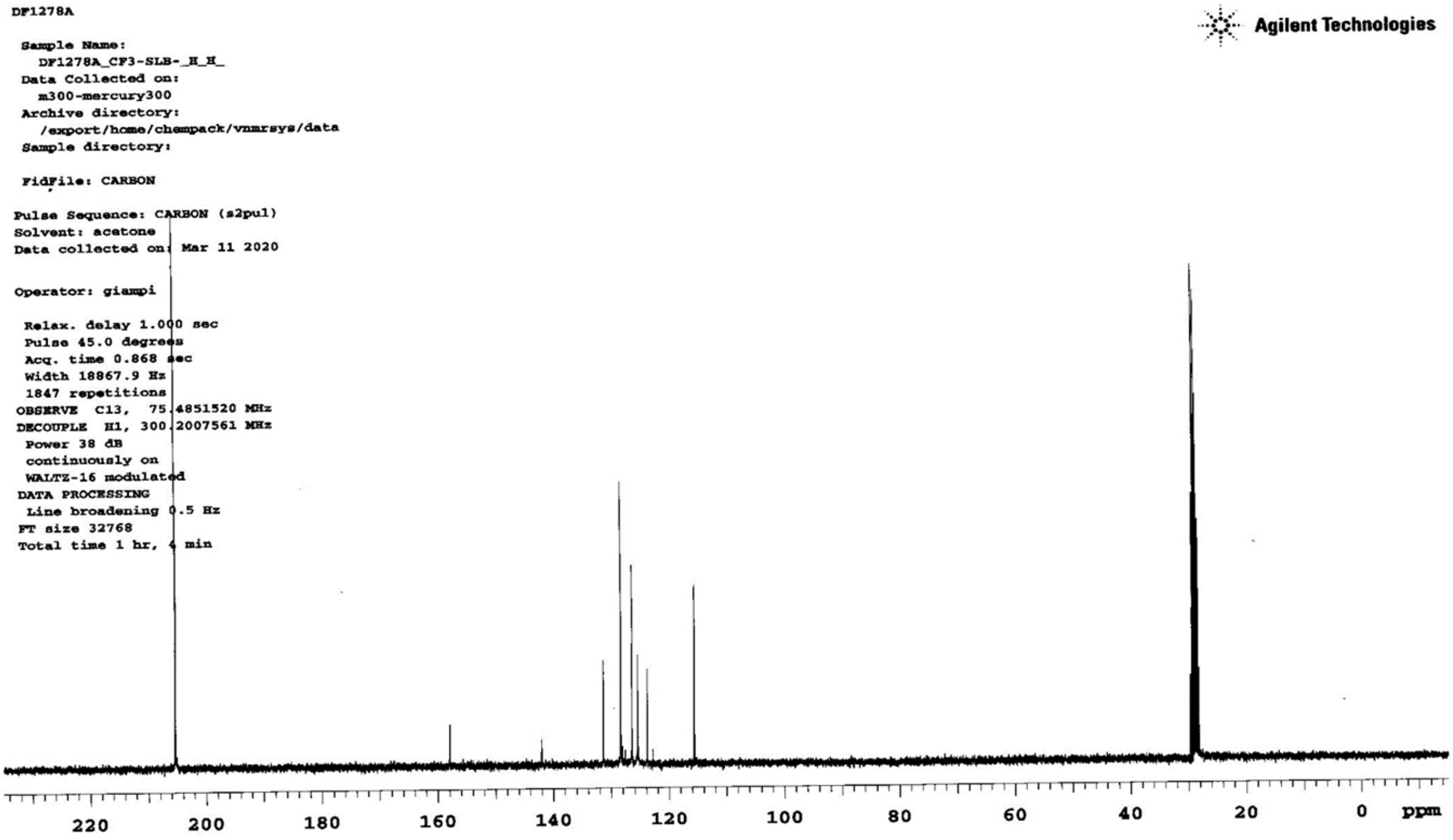
DF1278A
Sample Name:
DF1278A_CF3-SLB-_H_H_
Data Collected on:
m300-mercury300
Archive directory:
/export/home/chempack/vnmrsys/data
Sample directory:
FidFile: CARBON
Pulse Sequence: CARBON (s2pul)
Solvent: acetone
Data collected on: Mar 11 2020
Operator: giampi
Relax. delay 1.000 sec
Pulse 45.0 degrees
Acq. time 0.868 sec
Width 18867.9 Hz
1847 repetitions
OBSERVE C13, 75.4851520 MHz
DECOUPLE H1, 300.2007561 MHz
Power 38 dB
continuously on
WALTZ-16 modulated
DATA PROCESSING
Line broadening 0.5 Hz
FT size 32768
Total time 1 hr, 4 min
Agilent Technologies
220
200
180
160
140
120
100
80
60
40
20
0
ppm
